# Utilization of palliative care services and associated factors among cancer patients in Ethiopia: A systematic review and meta-analysis

**DOI:** 10.1371/journal.pone.0345299

**Published:** 2026-03-23

**Authors:** Sadik Abdulwehab, Frezer Kedir

**Affiliations:** 1 School of Nursing, Wollega University, Oromia, Ethiopia; 2 School of Nursing, Jimma University, Southwest Oromia, Ethiopia; Menzies School of Health Research: Charles Darwin University, AUSTRALIA

## Abstract

**Introduction:**

Palliative care is vital for cancer management in low- and middle-income countries like Ethiopia, but underutilization leads to unmanaged symptoms and reduced patient quality of life, and fragmented studies hinder evidence-based planning and policy development. This systematic review and meta-analysis aim to synthesize existing literature to estimate the utilization rate of Palliative care among cancer patients in Ethiopia and to identify key influencing factors.

**Review method and data sources:**

This study employed a systematic review and meta-analysis design to assess Palliative care utilization and its influencing factors among cancer patients in Ethiopia, sourcing evidence from various electronic databases until April 07, and studies published between 2015 and 2024 were included. The data was extracted from June 10–20 and analyzed from June 21–30, with report generation till July 27, 2025, using R software. Meta-analysis was performed using a random-effects model, with forest plots illustrating pooled prevalence and associated factors. Heterogeneity was assessed using the I² statistic, and study quality was evaluated by using a validated tool, the Joanna Briggs Institute Critical Appraisal Checklist.

**Results:**

A total of nine cross-sectional studies involving 2,839 cancer patients were included. The pooled Palliative care utilization rate was 42% (95% CI: 30%–54%). Educational attainment (pooled AOR = 2.57; 95% CI: 1.42–3.75) and male gender (pooled AOR = 5.58; 95% CI: 3.01–10.33) were factors significantly associated with Palliative care utilization.

**Conclusion:**

This review showed the Palliative care utilization rate was 42%. Palliative care utilization in Ethiopia remains insufficient, reflecting systemic, socioeconomic, and geographic inequities. Expanding access will require decentralization of services to reach rural communities, integration of Palliative care into primary healthcare, investment in workforce capacity, and improved patient and family awareness. Strengthening these areas is essential to ensure equitable, patient-centered, and sustainable Palliative care delivery in Ethiopia.

**PROSPERO registration number:**

CRD420251027739.

## Introduction

Cancer remains a significant public health challenge globally, with low- and middle-income countries (LMICs) experiencing a disproportionate burden [1–3]. LMICs often lack adequate healthcare infrastructure, leading to late-stage cancer diagnoses and limited treatment options, resulting in higher mortality rates compared to high-income countries [4].

The International Agency for Research on Cancer reported in 2020, 19.3 million new cancer cases and 10.0 million cancer-related deaths globally, 70% of these deaths occurring in low- and middle-income countries [5,6]. In Ethiopia, there were 80,034 new cancer cases and 54,698 cancer-related deaths in 2022 [7]. Currently, the World Health Organization (WHO) emphasizes the importance of comprehensive care approaches that go beyond curative treatment, incorporating Palliative care [8].

Palliative care is a patient-centered, multidisciplinary approach that aims to improve the quality of life of individuals with life-threatening illnesses through early identification and management of physical, psychosocial, and spiritual needs [9]. Despite its proven benefits, Palliative care access remains severely limited worldwide, with fewer than 15% of those in need receiving care due to service shortages, lack of trained personnel, and poor integration into health systems [10,11]. These challenges are especially severe in low- and middle-income countries, where limited awareness, inadequate training, lack of structured services, cultural beliefs, geographic barriers, and socioeconomic inequities reduce Palliative care utilization [11].

In sub-Saharan Africa, Palliative care services for cancer patients remain poorly integrated and underutilized [12]. In Ethiopia, the Ministry of Health recognizes the importance of Palliative care, but its implementation and utilization remain low. This results in unmanaged symptoms, psychological distress, and increased financial and healthcare burdens for patients and families [13–15].

There is a growing yet fragmented body of evidence on the factors influencing Palliative care utilization among cancer patients in Ethiopia, as some studies underscore the role of cultural beliefs and social stigma [16], while others highlight logistical challenges such as limited access to care and a shortage of trained personnel, reflecting inconsistencies in the literature [15]. Moreover, there is a noticeable lack of research specifically tailored to the unique Palliative care needs of Ethiopian cancer patients, particularly in areas like pain management, psychological support, and end-of-life care [17]. Compounding these challenges is the evident gap between knowledge and practice; healthcare providers often lack adequate training in evidence-based Palliative care, especially in rural regions where services are scarce [18].

Over the past two decades, Ethiopia has made significant strides in Palliative care by collaborating with the Ministry of Health, Non-Governmental Organizations, and International partners. Efforts include integrating Palliative care into the National Cancer Control Plan, establishing dedicated units, and gradually introducing oral morphine for pain management [16]. Training programs for health professionals have also been initiated to take steps toward recognizing Palliative care as a component of the health system [16,19].

Palliative care in Ethiopia faces challenges in scalability and accessibility, particularly in rural areas, where services are concentrated in urban hospitals like Addis Ababa, despite formal advancements [16]. Challenges include a limited workforce due to insufficient pre-service and in-service training, shortages in essential medications (e.g., opioids), weak community-healthcare facility linkages, and low public and provider awareness of Palliative care principles [10,20]. These issues are compounded by reliance on external donors and a lack of home-based or community-level service models [16,21].

Ethiopia has prioritized strengthening Palliative care as an essential component of Universal Health Coverage (UHC), aligning with Sustainable Development Goal 3(SDG3) and the national health sector strategies. Although Ethiopia set a target to integrate Palliative care and pain management services into at least 50% of public health facilities by 2020, recent studies show that these services remain limited, especially outside major urban centers. This gap between policy targets and actual service delivery highlights the urgent need to decentralize Palliative care, strengthen workforce capacity through pre-service and in-service training, expand community- and home-based delivery models, and better integrate Palliative care into primary healthcare to advance national UHC and SDG commitments [19,22,23].

The research on Palliative care utilization among cancer patients in Ethiopia is limited by fragmented and sometimes contradictory findings. There is no comprehensive synthesis of these studies using meta-analytic techniques to identify the most influential factors affecting Palliative care utilization in the Ethiopian context. A systematic review and meta-analysis are needed to summarize existing knowledge, quantify utilization rates, and identify consistent predictors across various settings. This evidence is crucial for national policy development and promoting equitable access to Palliative care for all.

## Methods

### Aim of the study

This systematic review and meta-analysis aim to estimate the utilization of Palliative care services and identify associated factors among cancer patients in Ethiopia to inform healthcare planning and improve Palliative care delivery.

### Design

This review employed a systematic review and meta-analysis design guided by the Preferred Reporting Items for Systematic Reviews and Meta-Analyses (PRISMA 2020) checklist [24]. Relevant studies on Palliative care utilization in Ethiopian cancer patients were identified through comprehensive database searches. Eligible studies were screened, and data were extracted using a structured form. Methodological quality was assessed using validated tools. Meta-analysis was conducted using R software, employing a random-effects model to estimate pooled proportions for utilization rates and associated factors. Heterogeneity was assessed using the I² statistic, and publication bias was evaluated using the Galbraith plot.

### Research question

This review investigates the extent of Palliative care service utilization among cancer patients in Ethiopia and identifies the socio-demographic, clinical, and system-level factors influencing it. The research question includes: What is the pooled prevalence of Palliative care utilization among cancer patients in Ethiopia? and What are the key factors associated with utilization?

### Inclusion and exclusion criteria

Included in the review were published and unpublished observational and interventional studies reporting on Palliative care utilization and/or its influencing factors among adult cancer patients in Ethiopia. Eligible studies were required to provide at least one of the following: (a) prevalence of Palliative care utilization, or (b) quantitative data on associated factors (e.g., sociodemographic, clinical, or system-level predictors). This approach ensured that studies could meaningfully contribute to either pooled prevalence estimates or factor analysis. Studies reporting demographic or service accessibility information without utilization outcomes were excluded because they did not address our research questions. Peer-reviewed articles, theses, dissertations, and relevant gray literature were considered without language or date restrictions. Excluded from the review were case reports, expert opinions, reviews, conference abstracts, and studies lacking data on Palliative care utilization or its determinants. Non-human studies were excluded because they do not contribute to understanding patient-level service utilization. Studies focusing primarily on non-Ethiopian patients were also excluded to preserve national representativeness and ensure that findings reflect the utilization patterns of the Ethiopian population within its healthcare system.

### Search strategy

**Fig 1:**
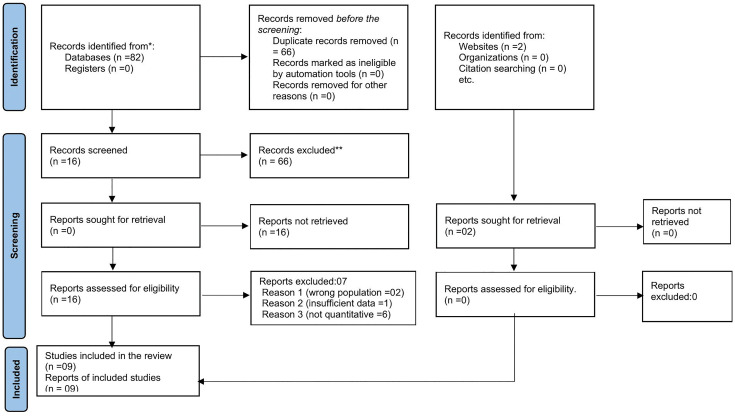
Flow chart diagram and PRISMA checklist describing the selection of studies for the systematic review and meta-analysis on Palliative care among cancer patients in Ethiopia, 2025.

A systematic search was conducted in electronic databases, including PubMed, Scopus, Web of Science, Google Scholar, African Journals Online (AJOL), and Ethiopian university repositories. Search terms included combinations of: “Palliative care,” “Palliative care utilization,” “cancer,” “oncology,” “Ethiopia,” and “associated factors.” Boolean operators and Medical Subject Headings (MeSH) terms were used where applicable (Table 1). Reference lists of selected articles were also manually screened. No restrictions on publication year and language were applied. Duplicate records were removed, and two independent reviewers screened the titles, abstracts, and full texts to ensure the inclusion of eligible studies. Discrepancies were resolved through discussion or consultation with each other. The database was searched for every article published on Palliative care utilization among cancer patients till June 10, 2025, and continued to update until we sent it for publication. The data was extracted from June 10–20 and later analyzed from June 21–30, and the report generation was completed by July 27, 2025 (Fig 1). We registered for this study with the CRD420251027739 registration number.

**Table 1:** Search strategy and retrieval summary on palliative care utilization among cancer patents in Ethiopia, 2025.

Database	Search Strategy (keywords/MeSH terms)	Records Retrieved	After Duplicates Removed	Full-texts Assessed	Studies Included
PubMed	(“palliative care”[MeSH] OR “palliative care utilization” OR “end-of-life care”) AND (“cancer” OR “oncology”) AND “Ethiopia”	24	18	5	3
Scopus	(TITLE-ABS-KEY (“palliative care” OR “end-of-life care”) AND TITLE-ABS-KEY (“cancer”) AND TITLE-ABS-KEY (“Ethiopia”))	14	10	4	2
Web of Science	(“palliative care” OR “end-of-life care”) AND (“cancer”) AND (“Ethiopia”)	12	9	3	1
CINAHL	(MH “Palliative Care” OR “end-of-life care”) AND (MH “Cancer” OR “Oncology”) AND Ethiopia	16	11	2	1
AJOL (African Journals Online)	“Palliative care” AND “cancer” AND “Ethiopia”	9	6	1	1
Google Scholar	Allintitle: “palliative care” AND “cancer” AND “Ethiopia”	16	12	1	1
Ethiopian University Repositories	“palliative care utilization” AND “cancer” AND “Ethiopia”	1	1	0	0
**Total**	82	65	16	9	

### Search outcomes

A total of 82 records were identified across databases: PubMed (24), CINAHL (09), Scopus (14), Web of Science (12), CINAHL (07), and Google Scholar (16). After removing duplicates and applying inclusion criteria, 16 full-text articles were assessed. Nine studies met all inclusion criteria and were included in the final analysis. Screening and data extraction were conducted independently by two reviewers, with discrepancies resolved through discussion (Fig 1).

### Data extraction

Based on the Joanna Briggs Institute methodology principles [25], a data extraction template was created. Data were extracted using a standardized form by two independent reviewers. Extracted information included: authors, year, study design, sample size, and region. Patient-level data included age, sex, cancer type, comorbidities, and performance status. Palliative care-related data captured types of services received (e.g., pain relief, spiritual care, psychological support), utilization frequency, patient satisfaction, and barriers to access. Treatment outcomes, mortality data, and predictors of service use were also collected. Authors were contacted for missing information when necessary (Table 2).

**Table 2:** Characteristics of included studies on Palliative care utilization and its influencing factors among cancer patients in Ethiopia, 2025.

Author & year	Title	Region/Setting	Design	Sample & Population	Characteristics	Types of PC Given	Outcome Measures	PC Delivery	Utilization Rate	Associated Factors with AOR,CI, P value	Limitations	Conclusions	Recommendations
Afessa et al. - 2024	Palliative care service utilization and associated factors among cancer patients at oncology units of public hospitals in Addis Ababa, Ethiopia	Addis Ababa (Tikur Anbessa & St. Paul)	Institution-based cross-sectional	404 adult cancer patients	63.4% aged 18–47, 56.7% female, 63.4% urban	Symptom management, psychological support, end-of-life care	PC utilization (35.4%), knowledge, attitude, satisfaction	Public hospital oncology units	35.4% [95% CI: 31.4–40.3%]	Education (college/university): AOR = 2.3, 95% CI: 1.15–4.61, p < 0.05 Living <23 km from facility: AOR = 1.8, 95% CI: 1.07–3.09, p < 0.05 Inability to read/write: AOR = 0.31, 95% CI: 0.14–0.67, p < 0.05 Low satisfaction with healthcare: AOR = 0.40, 95% CI: 0.23–0.72, p < 0.05 Treatment side effects: AOR = 0.41, 95% CI: 0.24–0.72, p < 0.05	Only urban settings; cross-sectional design	Low PC utilization; systemic/personal barriers	Enhance education, accessibility, and integration of PC
Amare et al. - 2023	The Prevalence of Cancer Patients Requiring Palliative Care and Its Associated Factors at St. Paul Hospital	St. Paul Hospital, Addis Ababa	Cross-sectional	301 admitted cancer patients	Mean age 42, 58.5% female, from various Ethiopian regions	Not directly delivered; need assessed via SPICT-LIS	10.6% needed PC, symptoms burden, performance decline	Inpatient oncology ward	10.6% (need identified)	Age > 61: AOR = 2.39, 95% CI: 0.34–16.55 Male gender: AOR = 5.31, 95% CI: 1.68–11.79, p = 0.001 Education (no formal education): Associated with higher PC need, p = 0.01 Region (e.g., SNNPR 25% vs Addis Ababa 0%): p < 0.0001 Marital status (married): More likely to need PC, p = 0.005	Single-center; tool-based assessment	High unmet need for PC among inpatients	Early PC screening; strengthen hospital-based services
**Fetene et al., 2024**	Factors affecting need and utilization of palliative care services among Ethiopian women in an oncology department	Hawassa University Comprehensive and Specialized Hospital, Sidama Region	Hospital-based cross-sectional study	121 women ≥18 years with breast cancer	Mean age 50, 58.7% rural, 50.4% primary schoo, majority stage 3 BC	Counseling, psychotherapy, pain treatment, chemotherapy	Utilization categorized as better or worse based on mean score	Provided by physicians &nurses in inpatient &outpatient units	59.5% worse utilization *(no CI reported)*	Rural residence (AOR = 11.82, *p* < 0.05)	Small sample size, single setting, no control group	More than half had poor PC utilization; rural residence was a major factor	Expand PC access and outreach in rural communities
Kebebew et al. - 2022	Hospital-based evaluation of palliative care among patients with advanced cervical cancer	Radiotherapy center, Specialized tertiary hospital, Ethiopia	Cross-sectional, hospital-based	385 women with advanced cervical cancer	Mean age: 52 years; 61.8% aged ≥50; 63.1% illiterate; 84.2% with income < $50/month	Pain treatment, symptom control (bleeding/discharge), limited counselling, spiritual, economic & home-based care	Knowledge, attitude, practice of PC; pain control; access to support	Mostly hospital-based, with some home visits	~90.3% received pain treatment, but only 56.3% had fair or complete relief	No AOR/CI/p-values reported for PC utilization or factors; analysis was descriptive	Excluded early-stage patients and those not attending the hospital; focus only on advanced cases	Most received some PC, but it was non-comprehensive; poor in education, spiritual and psychological support	Raise awareness of full PC scope; provide culturally appropriate communication on death & dying; ensure comprehensive PC at all healthcare levels
Lakew et al., 2015	Assessment of knowledge, accessibility and utilization of palliative care services among adult cancer patients	Tikur Anbessa Specialized Hospital, Addis Ababa	Institution-based cross-sectional study	384 adult cancer patients	Mean age 45.8, 62% married, 89.8% reported accessibility issues	Hospital counseling, community support, home-based care	Utilization based on basic PC service responses	Delivered through inpatient and outpatient departments	69% utilization (no CI reported)	Knowledge (AOR = 26.9, CI: 12.3–59); Little physical wellbeing (AOR = 3.1, CI: 1.96–4.9); Full social wellbeing (AOR = 1.7, CI: 1.01–2.8); Income $25–50 (AOR = 0.25, CI: 0.09–0.7); Single marital status (AOR = 55.4, CI: 1.2–2660.4)	Self-reporting, recall and social desirability bias	Knowledge and wellbeing influenced utilization; access remains limited	Introduce educational sessions, improve service awareness and support
Teklemariam et al., 2022	Perception about palliative care and factors influencing the likelihood of palliative care service utilisation	Tikur Anbessa Specialized Hospital, Addis Ababa	Facility-based cross-sectional study	304 adult cancer patients	Median age 56, 59.5% female, 56.9% rural, 48.4% no formal schooling	Chemotherapy, radiation, complaint therapy, supportive PC	Utilization determined via Likert scale adjusted score	PC services from hospital-based oncology team	42.8% utilization (no CI reported)	Income ≥ $52.35 (AOR = 2.36, CI: 1.37–4.06); Family members >2 (AOR = 2.28, CI: 1.02–5.13); Gov’t employee (AOR = 0.42, CI: 0.20–0.87); Formal schooling (AOR = 0.51, CI: 0.23–0.94)	Cross-sectional design limits causality; single facility	Utilization remains low; affected by income, family support, &employment status	Design targeted outreach for low-income and less-supported patients
Worku et al., 2017	Rehabilitation for cancer patients at Black Lion hospital, Addis Ababa, Ethiopia: a cross-sectional study	Black Lion Hospital, Addis Ababa	Cross-sectional study	388 adult cancer patients	Mean age 44, 68.6% female, 25% breast cancer, 20.6% colorectal	Rehabilitation, nutritional and psychosocial support	Service use recorded if received at least once	Rehabilitation services by hospital-based team	26% utilization (no CI reported)	Not specified in detail	Convenience sampling, missing CI and detailed factor	Low rehabilitation service utilization among patients	Enhance access, train staff, and increase resource availability
Bunare et al., 2022	Utilization of Rehabilitation Services and Associated Factors Among Adults With Cancer Diagnoses	Hawassa Comprehensive Specialized Hospital, Ethiopia	Institutional-based cross-sectional study	325 adults with cancer diagnoses selected via systematic sampling	Adults with cancer; both genders; assessed on ADL, social support, satisfaction	Cancer rehabilitation (physical, cognitive, emotional, social support)	Utilization defined as attending ≥1 rehabilitation service in the last year	Hospital-based rehabilitation services	33.2% (95% CI: 27.93–41.25)	- Male (AOR = 5.76; 95% CI: 2.60–12.75) – Urban residence (AOR = 2.56; 95% CI: 1.04–6.26) – Independent in ADLs (AOR = 2.68; 95% CI: 1.20–6.00) – Received education on rehab (AOR = 2.44; 95% CI: 1.21–4.91) – Strong social support (AOR = 2.10; 95% CI: 1.02–4.87) – Satisfaction with care (AOR = 3.21; 95% CI: 1.42–5.76)	Based on self-report and medical records; institutional scope may limit generalizability	Only one-third of patients utilized rehabilitation; several demographic and psychosocial factors influenced use	Improve awareness through patient education and strengthen supportive systems to enhance utilization
Aynalem et al., 2023	Utilization of palliative care and its associated factors among adult cancer patients in Hawassa University Comprehensive Specialized Hospital oncology center, Hawassa, Ethiopia, 2021	Hawassa University Comprehensive Specialized Hospital, Oncology Center	Institution-based cross-sectional study	180 adult cancer patients (≥18 years), randomly selected	66% were aged ≥50 years; income, education, and accessibility varied	Not explicitly listed (general palliative care services provided by the oncology center)	Categorized as “better” utilization based on structured questionnaire data	PC services delivered within oncology unit at the hospital	63% utilization (CI not explicitly provided)	- Age < 50 (AOR = 2.7; 95% CI: 1.13–6.63) – Education (Grade 9–12: AOR = 1.46; CI: 0.41–5.21; College + : AOR = 3.23; CI: 0.98–10.61) – Income >5,500 Birr (AOR = 2.7; CI: 0.51–5.76) – Good accessibility of PC (AOR = 2.99; CI: 1.21–3.28)	Limited sample size; cross-sectional design limits causal inference; CI not consistently precise	Two-thirds of patients had better PC utilization; older, less-educated, low-income, and rural patients had lower access	Improve PC information provision,especially for older and lesseducated patients;enhance accessibility in rural & suburban areas

### Organizing, summarizing, and reporting the results

Findings were organized into three main categories: (1) Demographic and clinical characteristics of cancer patients receiving Palliative care, (2) Utilization patterns of Palliative care services and the types provided, and (3) Predictors of Palliative care service use. Meta-analyses were conducted to pool estimates of Palliative care utilization rates and influencing factors. Results were reported in descriptive and tabular formats and illustrated using charts and figures. The PRISMA checklist guided the reporting process to ensure rigor and transparency.

### Quality appraisal

The methodological quality of the included studies was evaluated using the Joanna Briggs Institute (JBI) Critical Appraisal Checklist for Analytical Cross-Sectional Studies [25]. Each study was independently assessed by two reviewers across domains such as sample selection, outcome measurement, control of confounding variables, and statistical analysis. Studies scoring 7–8 “Yes” responses were considered low risk of bias, 4–6 as moderate risk, and 0–3 as high risk. Only low- and moderate-risk studies were included in the final synthesis (Table 3).

**Table 3:** JBI critical appraisal on Palliative care service utilization among cancer patients in Ethiopia, 2025.

Study No.	Afessa et al., 2024	Amare et al., 2023	Kebebew et al., 2022	Lakew et al., 2015	Teklemariam et al., 2022	Worku et al., 2017	Bunare et al., 2022	Aynalem et al., 2023	Fetene et al., 2024
Was there a clear statement of the aims of the research?	✔	✔	✔	✔	✔	✔	✔	✔	✔
Was the study design appropriate for the aims of the research?	✔	✔	✔	✔	✔	✔	✔	✔	✔
Was the sample representative of the population studied?	✔	✘	✔	✔	✔	✘	✔	✔	✔
Was the sample size adequate?	✔	✘	✘	✔	✔	✔	✘	✘	✘
Were the study subjects and the setting described in detail?	✘	✔	✔	✔	✔	✔	✔	✔	✔
Was the data collected in a reliable way?	✘	✔	✘	✘	✔	✔	✔	✔	✔
Were the statistical analyses used to assess the data appropriate?	✔	✔	✔	✔	✔	✔	✔	✔	✔
*Were the findings valid and applicable to the local context?*	✔	✔	✔	✔	✔	✔	✔	✔	✘
*Overall Quality*	6	6	6	7	8	7	7	7	6

Key: ✔ = Yes (Criterion met), ✘ = No (Criterion not met) and? = Unclear (Not adequately reported).

### Statistical analysis

Data analysis was performed using R software. Descriptive statistics, including frequencies, percentages, means, and standard deviations, were used to summarize study characteristics.

A meta-analysis was conducted using a random-effects model. A random-effects approach using Restricted Maximum Likelihood (REML) estimation was selected a priori due to anticipated clinical and methodological heterogeneity across studies. This approach estimates the mean of a distribution of true effects rather than assuming a single common effect size. Publication bias and small-study effects were explored using the Galbraith (radial) plot. Although alternative approaches, such as the Doi plot and LFK index, have been recommended for prevalence meta-analyses, a valid implementation could not be obtained using available R for the current dataset. In accordance with recent guidance, we employed REML estimation and applied Hartung-Knapp adjustments to compute more robust confidence intervals for pooled estimates and heterogeneity parameters [26,27]. Hartung–Knapp adjustments were applied to produce more conservative confidence intervals, particularly important given the small number of studies and high heterogeneity.

Heterogeneity was assessed using I² statistics, with substantial heterogeneity defined as I² > 50%. We also explored random-effects models for aggregate data as a sensitivity approach [28]. Given the expected variability in study populations, settings, and measurement approaches, pooled prevalence estimates were interpreted cautiously as descriptive aggregates rather than definitive national estimates. Sensitivity analyses were performed by excluding studies at high risk of bias or with small sample sizes. A leave-one-out sensitivity analysis was performed using a random-effects model using the Tau² value, which represents the estimated between-study variance under the random-effects model, reflecting the magnitude of true heterogeneity beyond sampling error. All findings were reported with 95% confidence intervals, with statistical significance set at p < 0.05. This reporting conforms with the PRISMA 2020 guidelines, and we have also considered emerging enhancements expected in PRISMA 2025 [24,29].

### Certainty of evidence assessment

The certainty of evidence was assessed using the Core GRADE approach, which provides a streamlined evaluation of overall certainty following meta-analysis, particularly suitable for observational studies.

### Ethical consideration

This review involved a secondary analysis of existing published studies, and therefore, ethical approval was not required. Ethical principles were upheld by ensuring all included studies had appropriate ethical clearance and informed consent. Proper citation and acknowledgment of original authorship were maintained throughout to ensure transparency and respect for intellectual property.

## Results

### Characteristics of the included studies

The included studies were all cross-sectional in design and conducted within various institutional settings across Ethiopia, primarily in public hospitals and specialized oncology centers [13,14,30–36]. Sample sizes ranged from 121 to 404 adult cancer patients, with participants mostly aged between 18 and 66 years and a majority being female [14,31,35]. Most studies recruited participants from urban hospitals in Addis Ababa, such as Tikur Anbessa and St. Paul’s [13,14,31,33–35], while others included patients from Hawassa and Sidama regions to reflect a more diverse geographic representation [30,32,36].

### Types of palliative care provided for cancer patients in Ethiopia

Across the reviewed studies, Palliative care services for cancer patients in Ethiopia varied in scope, delivery, and comprehensiveness. The most commonly reported components included pain and symptom management, psychological support, and end-of-life care [14,30]. In some cases, care was extended to include counseling, chemotherapy, radiation, and complaint-based supportive care [13,31]. At specialized units, such as those in Hawassa and tertiary hospitals, services incorporated psychotherapy, economic assistance, and limited spiritual support, although these were often inconsistently applied or underdeveloped [33,36]. Community and home-based care were mentioned in a few studies, but were not widespread or fully integrated [30,33]. Rehabilitation-focused studies highlighted nutritional, cognitive, emotional, and social support services, indicating efforts to expand Palliative care beyond clinical symptom control [32,34].

### Palliative care service delivery among cancer patients in Ethiopia

Palliative care delivery across the included Ethiopian studies was predominantly institutional, with services administered within hospital-based oncology units or inpatient and outpatient departments [13,14,30].Two studies [30,33] referenced community or home-based services. Palliative care delivery models largely lacked specialized structures or referral systems and were primarily focused on curative or hospital-based treatment settings, rather than holistic end-of-life care. In some instances, such as in Hawassa and Addis Ababa, the delivery mechanisms were limited to inpatient hospital units, restricting accessibility for rural populations and those with mobility or economic challenges [31,36].

### Palliative care service measures

The studies included in this review utilized a range of outcome measures to assess Palliative care utilization and effectiveness among cancer patients in Ethiopia. Most commonly, outcomes focused on the rate of Palliative care utilization are typically reported as a percentage of participants receiving or needing Palliative care services [14,36]. Several studies also assessed patient knowledge, attitude, and satisfaction toward Palliative care as indirect indicators of service uptake and perception [14,30]. A functional performance decline, symptom burden, and the need for Palliative care services were evaluated as Palliative care utilization [35]. Other studies categorized utilization levels based on structured questionnaire scores as better or worse [31,36]. For rehabilitation-related services, outcomes included attendance at rehabilitation sessions, level of independence in activities of daily living (ADLs), and patient satisfaction with care [32,34]. Despite this diversity, the lack of standardized and validated outcome measures across studies made cross-comparison challenging [13,14,30–36].

Palliative care utilization was measured variably across studies, including receipt of at least one Palliative care service, referral to a Palliative care unit, self-reported utilization, or classification based on structured questionnaire scores. Although this variability reflects real-world practice, it contributes to between-study heterogeneity.

### Palliative care service utilization rate among cancer patients in Ethiopia

**Fig 2:**
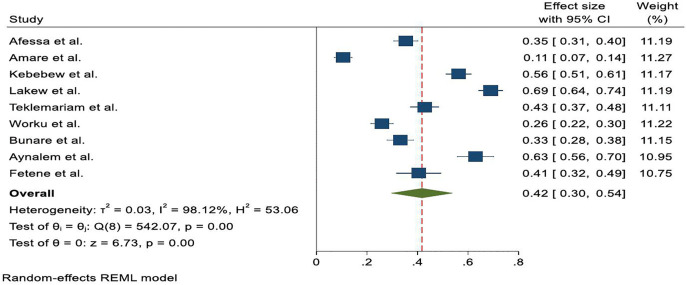
Forest plot shows the pooled utilization rate of Palliative care service among cancer patients in Ethiopia, systematic review and meta-analysis, 2025.

Palliative care utilization rates among cancer patients in Ethiopia showed marked variability across the included studies, reflecting heterogeneity in service availability, health system readiness, and patient-level factors. The reported prevalence ranged from 10.6% in Amare et al. (2023 to 69.0% in Lakew et al. (2015. The random-effects meta-analysis, including nine studies, estimated a pooled prevalence of 42% (95% CI: 30%–54%)**.** Given the substantial heterogeneity observed across studies, this pooled estimate should be interpreted as a descriptive aggregate rather than a definitive prevalence (Fig 2).

### Subgroup analysis

**Fig 3:**
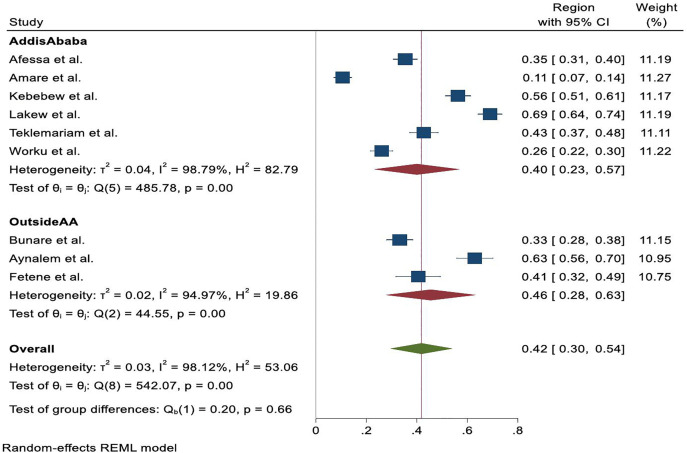
Forest plot indicating sub‐analysis of utilization of Palliative care based on region among cancer patients in Ethiopia, systematic review and meta-analysis, 2025.

To investigate potential sources of heterogeneity, subgroup analysis was conducted by geographic region. In Addis Ababa, the pooled prevalence of palliative care utilization was 39.0% (95% CI: 23%–57%), based on six studies, while outside Addis Ababa, the pooled prevalence was slightly higher at 46.0% (95% CI: 28%–63%), based on three studies. Although utilization rates appeared somewhat higher outside Addis Ababa, the differences were not statistically significant, suggesting that regional location alone may not account for the observed variation. Heterogeneity remained high within both subgroups (I² = 98.79% for Addis Ababa; I² = 94.97% for outside Addis Ababa) (Fig 3). All pooled analyses demonstrated substantial heterogeneity (I² > 90%), indicating considerable variability across studies. Consequently, pooled estimates should be interpreted as indicative rather than definitive national values.

**Fig 4:**
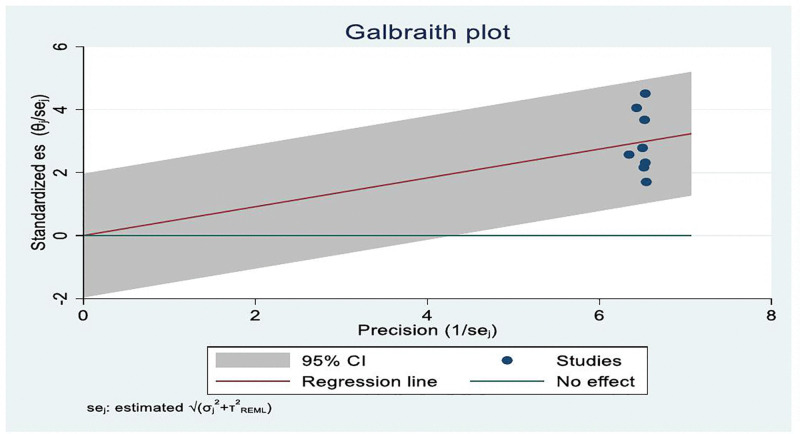
Galbraith plot shows pooled utilization rate of Palliative care service among cancer patients in Ethiopia, systematic review and meta-analysis, 2025.

The presence of publication bias was visually assessed using a funnel plot. The Galbraith plot did not reveal marked asymmetry or influential outliers, suggesting no strong evidence of publication bias among the included studies. However, in this meta-analysis, there is no strong evidence of publication bias noted in the data summary (Fig 4).

### Sensitivity analysis

A leave-one-out sensitivity analysis was performed using a random-effects model (Tau² = 0.0067) to evaluate the influence of individual studies on the overall pooled estimate. The analysis revealed only slight variation in the pooled effect size when each study was excluded one at a time, with estimates ranging from 0.380 to 0.457. The confidence intervals consistently overlapped, suggesting that no single study exerted a significant influence on the overall findings (Table 4).

**Table 4:** Influence of each study on pooled effect size on palliative care service utilization among cancer patients in Ethiopia, 2025.

Omitted Study	Region	Pooled Effect Size	95% CI Lower	95% CI Upper
Afessa et al., 2024	Addis Ababa	0.423	0.363	0.483
Amare et al., 2023	Addis Ababa	0.457	0.397	0.517
Kebebew et al., 2022	Addis Ababa	0.396	0.336	0.456
Lakew et al., 2015	Addis Ababa	0.380	0.320	0.440
Teklemariam et al., 2022	Addis Ababa	0.414	0.354	0.474
Worku et al., 2017	Addis Ababa	0.436	0.375	0.496
Bunare et al., 2022	Hawassa	0.426	0.366	0.486
Aynalem et al., 2023	Hawassa	0.390	0.331	0.450
Fetene et al., 2024	Hawassa	0.416	0.357	0.476

### Factors associated with palliative care utilization among cancer patients in Ethiopia

Included studies varied in the way they reported associations between predictors and Palliative care utilization. For studies that did not provide adjusted or crude odds ratios, raw frequency data were extracted where possible and used to compute crude odds ratios with 95% CIs. When raw data were insufficient for calculation, findings were narratively synthesized and described qualitatively. This ensured that all eligible studies contributed to the review, while avoiding potential bias from imputing unavailable estimates. Predictors reported by fewer than two studies with comparable effect measures were synthesized narratively and interpreted cautiously.

Factors associated with Palliative care utilization among cancer patients were assessed across the included articles in Ethiopia. Given the analysis of multiple factors, results should be interpreted cautiously, as the risk of type I error increases with multiple comparisons. Age was identified in one study as influencing Palliative care utilization, with patients under 50 years more likely to use Palliative care services by Aynalem et al. [36](AOR = 2.7, 95% CI: 1.13–6.63). Similarly, experiencing treatment side effects was reported in another study to be inversely associated with Palliative care utilization (Afessa et al., 14); AOR = 0.41, 95% CI: 0.24–0.72, p < 0.05). These findings are based on individual studies and interpreted cautiously.

**Fig 5:**
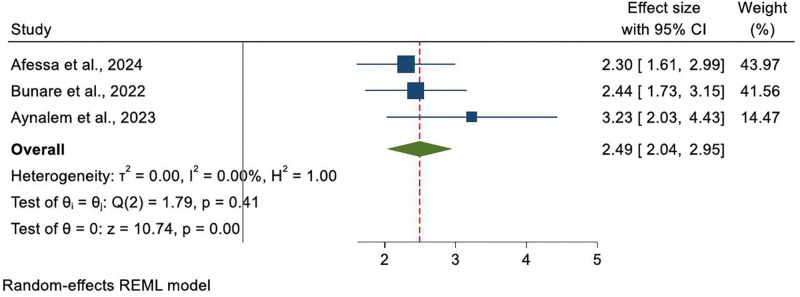
Forest plot shows the association of educational status and Palliative care service among cancer patients in Ethiopia, systematic review and meta-analysis, 2025.

Educational status appears to have a mixed influence. Higher education (college or university level) was positively associated with Palliative care use in several studies [14,32,36]. Pooled effect size for the association between educational status and Palliative care utilization among cancer patients in Ethiopia was found to be 2.49(CI: 2.04–2.95) (Fig 5).

**Fig 6:**
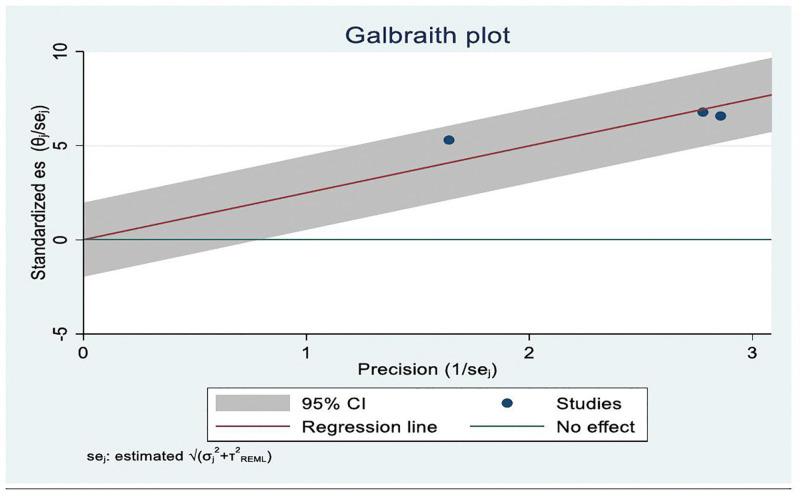
Galbraith plot shows the association of educational status and Palliative care service utilization among cancer patients in Ethiopia, systematic review and meta-analysis, 2025.

The study found that the Galbraith plot did not reveal marked asymmetry or influential outliers, suggesting no strong evidence of publication bias among the included studies (Fig 6).

**Fig 7:**
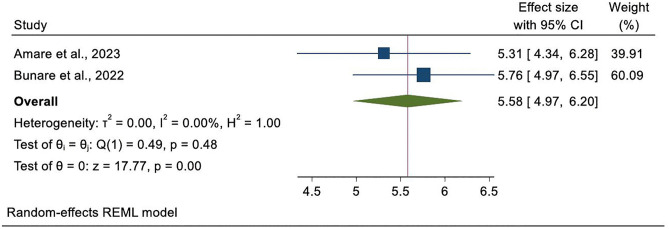
Forest plot shows the association of educational status and Palliative care service utilization among cancer patients in Ethiopia, systematic review and meta-analysis, 2025.

Gender was identified as a significant factor influencing Palliative care utilization among cancer patients in Ethiopia. Two studies [23,26] demonstrated a strong positive association between being male and higher service use. Bunare et al. [32] reported that male patients had markedly greater odds of utilizing Palliative care compared to females (AOR = 5.76; 95% CI: 2.60–12.75), while Amare et al. [35] found a similar association (AOR = 5.31; 95% CI: 1.68–11.79). When the findings from these two studies were pooled, the combined effect indicated that male patients were significantly more likely to access Palliative care services than their female counterparts (pooled AOR = 5.58; 95% CI: 4.97–6.20) (Fig 7).

A forest plot was generated to display the individual and pooled adjusted odds ratios for the association between sex and Palliative care utilization. Because the pooled analysis was based on only two studies, assessment of publication bias using funnel plots or statistical tests was not performed.

Access to healthcare services was identified as an important determinant of Palliative care utilization. One study reported that patients living within 23 kilometers of a healthcare facility had higher odds of using Palliative care services (Afessa et al.; AOR = 1.80) [14]. while another study found that patients with good accessibility to services were more likely to utilize Palliative care (Aynalem et al; AOR = 2.99) [36].

Satisfaction with care emerged as an important determinant of Palliative care utilization. Bunare et al. [32] reported that satisfied patients were significantly more likely to use services (AOR = 3.21; 95% CI: 1.42–5.76), while Afessa et al. [14] similarly found a positive association (AOR = 1.40; 95% CI: not reported); however, this estimate was not included in the meta-analysis because the confidence interval or standard error was not reported.

In addition, family and social support were consistently linked with higher service use. Teklemariam et al. [13] reported that patients with stronger family involvement had greater odds of utilizing services (AOR = 2.28; 95% CI: 1.02–5.13), and Bunare et al. [32] reported that patients with strong social support had greater odds of utilizing services (AOR = 2.10; 95% CI: 1.02–4.87).

Using the Core GRADE framework, the certainty of evidence for pooled Palliative care utilization and associated factors was rated as low to very low, primarily due to high heterogeneity, cross-sectional study designs, and inconsistency across studies. Bayesian meta-analysis was not performed due to the small number of studies and limited data for robust estimation. Although funnel plots and Egger’s tests were performed, the small number of studies (<10 per factor) limits their reliability; thus, the absence of detected publication bias does not confirm that bias is absent.

Given the analysis of multiple factors, results should be interpreted cautiously, as the risk of type I error increases with multiple comparisons. Overall, these findings indicate that sociodemographic factors, economic capacity, healthcare accessibility, patient satisfaction, and social support play important roles in influencing Palliative care utilization among Ethiopian cancer patients and are summarized in the table as a summary of factors associated with Palliative care Utilization among Cancer patients in Ethiopia (Table 5).

**Table 5:** Summary of factors associated with Palliative care utilization among cancer patients in Ethiopia.

Factor	No. of Studies	Contributing Studies (Composite)	Pooled OR	95% CI	I² (%)	Interpretation
**Educational status (college/university vs. lower)**	3	Afessa et al. (2024); Lakew et al. (2015); Aynalem et al. (2023)	2.49	2.04–2.95	0.0	Higher educational attainment significantly increases palliative care utilization
**Male sex**	2	Amare et al. (2023); Bunare et al. (2022)	5.58	4.97–6.20	Not calculated*	Male patients were substantially more likely to utilize supportive and palliative-related services
**Higher income**	2	Teklemariam et al. (2022); Aynalem et al. (2023)	2.69	1.23–5.90	High†	Greater economic capacity facilitates access to palliative care
**Good accessibility/ shorter distance to facility**	2	Afessa et al. (2024); Aynalem et al. (2023)	2.24	1.52–3.29	Moderate†	Proximity to health facilities significantly improves utilization
**Satisfaction with healthcare services**	2	Afessa et al. (2024); Bunare et al. (2022)	1.96	1.30–2.95	Low†	Patient satisfaction is positively associated with palliative care utilization
**Family and social support**	2	Teklemariam et al. (2022); Bunare et al. (2022)	2.18	1.29–3.68	Low†	Strong family and social support promote service uptake

* Heterogeneity (I²) was not calculated for factors pooled from fewer than three studies, as such estimates are statistically unreliable.

† Heterogeneity estimates should be interpreted cautiously due to variability in study design, outcome definitions, and measurement tools.

### Certainty of evidence assessment

The certainty of evidence was assessed using the Core GRADE approach, which provides a streamlined evaluation of overall certainty following meta-analysis, particularly suitable for observational studies (Table 6).

**Table 6:** Certainty of evidence assessment using core GRADE for palliative care utilization among cancer patients in Ethiopia, 2025.

Outcome	No. of Studies	Study Design	Risk of Bias	Inconsistency (Heterogeneity)	Imprecision	Publication Bias	Overall Certainty (Core GRADE)	Justification
**Pooled prevalence of palliative care utilization**	9	Cross-sectional	Serious	Serious (high I²)	Serious	Not formally assessed	**Low**	Observational design, high heterogeneity across regions and settings, variation in measurement of utilization
**Education level and PC utilization**	5	Cross-sectional	Serious	Serious	Serious	Not assessed	**Very Low**	Inconsistent effect sizes, wide confidence intervals, self-reported exposure
**Residence (urban/rural) and PC utilization**	4	Cross-sectional	Serious	Serious	Serious	Not assessed	**Very Low**	Substantial variability between studies and settings
**Income and PC utilization**	4	Cross-sectional	Serious	Serious	Serious	Not assessed	**Very Low**	Imprecise estimates and inconsistent direction of association
**Accessibility to PC services**	3	Cross-sectional	Serious	Moderate	Serious	Not assessed	**Low**	Consistent direction of effect but limited number of studies and cross-sectional design

## Discussion

This systematic review and meta-analysis synthesized evidence from nine cross-sectional studies assessing Palliative care service utilization among adult cancer patients in Ethiopia [13,14,30–36]. The overall findings demonstrate that Palliative care utilization remains with pooled estimates indicating that only 42% of eligible patients accessed services across the included studies and unevenly distributed across the country [13,14,30–36]. Despite the growing need for palliative services due to the increasing cancer burden, many patients continue to lack access to adequate care, with significant disparities observed between regions and institutions [14,31].

The majority of included studies were conducted in well-resourced urban tertiary hospitals, particularly in Addis Ababa [13,14,30,34]and the Sidama region [31,32,36]. This concentration reflects that most Palliative care services in Ethiopia are hospital-based and primarily urban-centered, with minimal integration into community or home-based care systems [14,33,36]. Consequently, the reported utilization rates likely reflect urban populations with better access to healthcare infrastructure, while rural and underserved populations may be underrepresented. These findings highlight that access disparities, including geographic location and facility resources, are important determinants of Palliative care utilization in Ethiopia [16].

The reviewed articles indicate that Palliative care services in Ethiopia have largely focused on pain and symptom management, psychological support, and end-of-life care, while broader services such as counseling, rehabilitation, and spiritual support are inconsistently provided or underdeveloped [31–33]. Although there has been notable progress in patient care over the past two decades, the integration of psychological, social, and spiritual support remains limited, with current services often providing insufficient medical, psychosocial, and financial support [37,38]. These findings suggest that Palliative care in Ethiopia is primarily hospital- and symptom-focused, and that gaps remain in delivering a fully comprehensive, patient-centered approach addressing all domains of patient well-being.

In terms of measurement, studies employed varied outcome indicators, some based on direct utilization rates, others relying on proxy measures such as patient knowledge, attitudes, and satisfaction [13,30,32]. This lack of standardization underscores the need for a more integrated, equitable, and standardized Palliative care system in Ethiopia to meet the growing needs of cancer patients nationwide.

This review revealed substantial variability in Palliative care utilization across studies, ranging from 10.6% to 69.0%, with very high heterogeneity. These findings suggest that differences in service availability, health system readiness, and patient-level factors strongly influence utilization. Subgroup analysis by region showed slightly higher utilization outside Addis Ababa (46.0%) compared to studies conducted in the capital (39.0%), indicating that geographic location alone does not fully account for the observed variation. The high heterogeneity likely arises from differences in outcome definitions, institutional capacity, urban–rural disparities, study populations, and availability of Palliative care services. The persistence of high heterogeneity in both subgroups highlights the complex interplay of institutional capacity, health workforce distribution, patient awareness, and socio-cultural perceptions of Palliative care in shaping access and utilization patterns.

The review showed that the pooled prevalence of Palliative care utilization among cancer patients in Ethiopia was found to be 42% (95% CI: 30% to 54%), which is higher than study done both in Kenya [39] and Uganda’s [40] which accounts 10%, Global & LMICs Context accounts 34.43% [41], largely due to improvements in healthcare infrastructure, growing public awareness, and better integration of services [42,43]. The high heterogeneity likely arises from differences in outcome definitions, institutional capacity, urban–rural disparities, study populations, and availability of Palliative care services. These findings underscore that utilization patterns are highly context-specific rather than uniform across Ethiopia.

Higher education (college or university level) was positively associated with Palliative care use in several studies [14,32,36] indicating that individuals with higher educational attainment were significantly more likely to utilize Palliative care services compared to their less educated counterparts, which is similar to study done in Uganda [44], Kenya [39], Nigeria [45], and Norway [46]. The association may reflect that individuals with higher education generally have greater health literacy, stronger communication skills, and increased autonomy in healthcare decision-making [47], which can facilitate engagement with Palliative care services.

The review article showed that gender has emerged as a significant factor influencing the utilization of Palliative care services among cancer patients in Ethiopia [32,35]. The gender estimate is based on only two studies and should therefore be interpreted cautiously. Male patients were more likely to use Palliative care services compared to their female counterparts, which is similar to the study done in Uganda [44], Kenya [39], and African countries [18]. The unusually high odds ratio may be explained by socio-cultural and healthcare factors: in patriarchal societies, men are often the primary decision-makers, have greater autonomy, and face fewer barriers in seeking healthcare. Women, on the other hand, may encounter multiple constraints, including caregiving responsibilities, cultural restrictions on mobility, financial dependency, and limited decision-making power. These structural and social barriers may substantially reduce women’s access to Palliative care, contributing to the observed strong association.

Higher income was linked to increased utilization of Palliative care [13,30]. It was reported that patients with adequate knowledge about Palliative care services were vastly more likely to utilize Palliative care, which is similar to a study done in Africa [18], Nigeria [48], Kenya [39], Uganda [49]. This pattern likely reflects that wealthier individuals can more readily afford healthcare-related expenses and may have greater awareness of the benefits of Palliative care [50], facilitating utilization.

The reviewed studies have shown that individuals living closer to healthcare facilities are more likely to use Palliative care services [14,36]. This finding is similar to studies done in Nigeria [51], Cameron [52], and Portugal [53]. Although detailed geographic mapping was not available, studies consistently reported that shorter travel distances or better accessibility to healthcare facilities were associated with higher Palliative care utilization. This suggests that geographical proximity and ease of access play critical roles in ensuring that patients can receive timely Palliative care, highlighting the need for better service distribution and infrastructure in underserved areas.

Several studies indicated that higher patient satisfaction was associated with increased utilization of Palliative care services [14,32], consistent with findings from Kenya [54], Sweden [55], and Norway [56]. This relationship likely reflects that high-quality, patient-centered care enhances trust and engagement, thereby encouraging patients to access essential services such as Palliative care.

The reviewed article showed that when family members and social networks are involved, patients are more likely to access and benefit from Palliative care [13,32], which is similar to studies done in Uganda [57], South Africa [58], and Kenya [48]. This is due to emotional support, assistance with decision-making, resource mobilization, and advocacy from family members. Healthcare systems should engage families by providing education on the benefits of palliative services, training healthcare providers to communicate effectively with both patients and families, and offering support resources for family caregivers.

### Strengths and limitations

This systematic review and meta-analysis offer several notable strengths. It is the first of its kind to provide a pooled national estimate of Palliative care utilization among cancer patients in Ethiopia, drawing on studies from diverse geographic regions, including both urban and semi-rural settings. The review adhered to rigorous methodological standards, following the PRISMA guidelines to ensure transparency and reliability. However, the study also has limitations. All included studies employed cross-sectional designs, limiting the ability to establish causal relationships. Additionally, variations in outcome measures across studies hindered comparability, and the scarcity of data from rural and community-based settings may have led to an underrepresentation of the most underserved populations. As most included studies were hospital-based and concentrated in urban referral centers, the findings may not fully reflect Palliative care realities in rural and community settings across Ethiopia. Meta-regression was not performed due to the small number of included studies, which would limit statistical power and produce unstable estimates. No formal correction for multiple comparisons was applied due to the exploratory nature of the factor analyses. The limited number of studies reporting gender-specific outcomes constrained our ability to draw robust conclusions regarding differences between male and female patients. This study includes data only from Addis Ababa and Hawassa, which may not represent all regions of Ethiopia. As a result, the findings might not fully reflect regional variations in the studied outcomes.

## Conclusion and recommendation

This systematic review and meta-analysis found that the utilization of Palliative care services among cancer patients in Ethiopia is moderate but overall insufficient, with a pooled prevalence of 42%. The findings highlight significant inequities in service use, with disparities observed by gender, education, income, and geographic location, indicating that rural and underserved populations have more limited access compared to urban residents. Critical system-level barriers were identified, including shortages of trained healthcare personnel, limited community-based services, and weak integration of Palliative care into the broader healthcare system. While progress has been made in areas such as pain and symptom management, essential components of comprehensive care, including psychosocial and spiritual support, remain underdeveloped. To enhance Palliative care utilization and ensure equitable, patient-centered services across Ethiopia, efforts should focus on: (1) decentralizing Palliative care delivery to reach rural and underserved populations, (2) investing in workforce development and training for healthcare providers, (3) integrating Palliative care into primary healthcare and standard cancer care pathways, (4) strengthening health policies and infrastructure to support service delivery, and (5) improving patient and community health literacy regarding Palliative care. Implementing these strategies can help reduce disparities and improve the overall accessibility, quality, and comprehensiveness of Palliative care services in Ethiopia.

**What is known**

- Palliative care is vital for cancer management but is limited in Ethiopia, especially in rural and underserved regions.
- Utilization varies due to differences in infrastructure, trained staff, and awareness among patients and providers.
- Cultural beliefs, low health literacy, financial barriers, and poor service integration hinder access and delivery.

**What is New?**

- First pooled national prevalence estimate (40.2%) of Palliative care utilization among cancer patients in Ethiopia.
- Identifies specific demographic and socioeconomic predictors of Palliative care use.
- Reveals the underdevelopment of community-based and home-based Palliative care models.

## Supporting information

S1 File PRISMA checklist for the systematic review and meta-analysis on Palliative care among cancer patients in Ethiopia, 2025. (DOCX)

S2 File Search Strategy and Retrieval Summary for the systematic review and meta-analysis on Palliative care among cancer patients in Ethiopia, 2025. (DOCX)

## References

[1] Amenu A, Aynalem A, Borie YA, Jemebere W, Molla E, Samuel B, et al. Determinants of unmet physical and psychological supportive care needs among adult cancer patients in Southern Ethiopia. BMJ Support Palliat Care. 2023;14(e2):e004606. doi: 10.1136/spcare-2023-004606 37816593 PMC11671935

[2] Bawden A, Health AB, Correspondent, Social Affairs. Global cancer cases to rise by more than 75% by 2050, WHO predicts. The Guardian [Internet]. 2024 [cited 2025 Apr 11]. Available from: https://www.theguardian.com/society/2024/feb/01/global-cancer-cases-to-rise-by-more-than-75-by-2050-who-predicts

[3] Pramesh CS, Badwe RA, Bhoo-Pathy N, Booth CM, Chinnaswamy G, Dare AJ, et al. Priorities for cancer research in low- and middle-income countries: a global perspective. Nat Med. 2022;28(4):649–57. doi: 10.1038/s41591-022-01738-x 35440716 PMC9108683

[4] Shah SC, Kayamba V, Peek RM, Heimburger D. Cancer control in low- and middle-income countries: is it time to consider screening? J Glob Oncol. 2019;5:1–8. 10.1200/JGO.18.00200 PMC6452918 30908147

[5] Sung H, Ferlay J, Siegel RL, Laversanne M, Soerjomataram I, Jemal A. Global cancer statistics 2020: GLOBOCAN estimates of incidence and mortality worldwide for 36 cancers in 185 countries. CA Cancer J Clin. 2021;71(3):209–49. 33538338 10.3322/caac.21660

[6] The Global Cancer Burden. The Global Cancer Burden [Internet]. 2020 [cited 2025 Apr 11]. Available from: https://www.cancer.org/about-us/our-global-health-work/global-cancer-burden.html

[7] Beneberu M, Teshale G, Demissie KA, Dellie E, Jejaw M, Atnafu A. Patient-centeredness and determinant factors of palliative care service for adult cancer patients in public hospitals of Addis Ababa, Ethiopia, 2024: cross-sectional mixed method study. BMC Palliative Care. 2025;24:57. 40055674 10.1186/s12904-025-01694-6 PMC11887271

[8] World Health Statistics 2020. World health statistics 2020: monitoring health for the SDGs, sustainable development goals [Internet]. 2020 [cited 2025 Apr 11]. Available from: https://www.who.int/publications/i/item/9789240005105

[9] Hannon B, Zimmermann C, Knaul FM, Powell RA, Mwangi-Powell FN, Rodin G. Provision of palliative care in low- and middle-income countries: overcoming obstacles for effective treatment delivery. J Clin Oncol. 2016;34(1):62–8. 26578612 10.1200/JCO.2015.62.1615

[10] Negasa EH, Human SP, Roro AG. Challenges in Palliative care provision in Ethiopia: an exploratory qualitative study. J Pain Res. 2023;16:3405–15. doi: 10.2147/JPR.S415866 37817758 PMC10561620

[11] Knaul FM, Farmer PE, Krakauer EL, De Lima L, Bhadelia A, Jiang Kwete X, et al. Alleviating the access abyss in palliative care and pain relief: an imperative of universal health coverage: the Lancet Commission report. Lancet. 2018;391(10128):1391–454. 29032993 10.1016/S0140-6736(17)32513-8

[12] Downing JD, Marston J, Selwyn C, Ross-Gakava L. Developing children’s palliative care in Africa through beacon centres: lessons learnt. BMC Palliat Care. 2013;12:8. doi: 10.1186/1472-684X-12-8 23419095 PMC3584905

[13] Teklemariam MF, Addise M, Asrat G, Gufue ZH. Perception about palliative care and factors influencing the likelihood of palliative care service utilisation among adult cancer patients in Ethiopia. Eur J Cancer Care (Engl). 2022;31(6):e13735. doi: 10.1111/ecc.13735 36221904

[14] Afessa N, Birhanu D, Negese B, Tefera M. Palliative care service utilization and associated factors among cancer patients at oncology units of public hospitals in Addis Ababa, Ethiopia. PLoS One. 2024;19(3):e0294230. doi: 10.1371/journal.pone.0294230 38483983 PMC10939243

[15] Abate Y, Solomon K, Azmera YM, de Fouw M, Kaba M. Barrier analysis for continuity of palliative care from health facility to household among adult cancer patients in Addis Ababa, Ethiopia. BMC Palliat Care. 2023;22(1):57. doi: 10.1186/s12904-023-01181-w 37173667 PMC10175902

[16] Aregay A, O’Connor M, Stow J, Ayers N, Lee S. Palliative care in Ethiopia’s rural and regional health care settings: a qualitative study of enabling factors and implementation challenges. BMC Palliat Care. 2023;22(1):156. doi: 10.1186/s12904-023-01283-5 37845671 PMC10580684

[17] Getie A, Wondmieneh A, Bimerew M, Gedefaw G, Demis A. Knowledge on palliative care and associated factors among nurses in Ethiopia: a systematic review and meta-analysis. Pain Res Manag. 2021;2021:5557947. doi: 10.1155/2021/5557947 33986898 PMC8093038

[18] Agom DA, Onyeka TC, Iheanacho PN, Ominyi J. Barriers to the provision and utilization of palliative care in Africa: a rapid scoping review. Indian J Palliat Care. 2021;27(1):3–17. doi: 10.4103/IJPC.IJPC_355_20 34035611 PMC8121217

[19] Aregay A, O’Connor M, Stow J, Ayers N, Lee S. Measuring and exploring the barriers to translating palliative care knowledge into clinical practice in rural and regional health-care settings. Palliat Support Care. 2023;:1–10. doi: 10.1017/S1478951523000755 37387249

[20] Aregay A, O’Connor M, Stow J, Ayers N, Lee S. Perceived policy-related barriers to palliative care implementation: a qualitative descriptive study. Palliat Care Soc Pract. 2023;17. doi: 10.1177/26323524231198542 37706166 PMC10496462

[21] Lynn A. Challenges and opportunities in expanding Palliative care access in Ethiopia. J Palliat Care Med. 2023;13(10):1–4.

[22] Reid E, Abathun E, Gebre N, Harding R, Lorenz K, Hauser J, et al. It takes a village: bringing Palliative care to Ethiopia. J Palliat Med. 2024;27(7):842–5. doi: 10.1089/jpm.2023.0632 38990601

[23] NCCP Ethiopia Final | PDF [Internet]. [cited 2025 Sep 4]. Available from: https://www.scribd.com/document/775252756/NCCP-Ethiopia-Final?utm_source=chatgpt.com

[24] Page MJ, McKenzie JE, Bossuyt PM, Boutron I, Hoffmann TC, Mulrow CD, et al. The PRISMA 2020 statement: an updated guideline for reporting systematic reviews. BMJ. 2021;372:n71. 10.1136/bmj.n71 PMC8005924 33782057

[25] JBI Manual for Evidence Synthesis. JBI Manual for Evidence Synthesis - JBI Global Wiki [Internet]. 2024 [cited 2025 Apr 12]. Available from: https://jbi-global-wiki.refined.site/space/MANUAL

[26] Justo M, Askin H, Mehta D, Benharash P. Pooled perspectives: critical considerations for the modern meta-analysis. Surg Open Sci. 2025;25:5–7. doi: 10.1016/j.sopen.2025.03.005 40213084 PMC11979908

[27] Random-effects meta-analysis methods for Intervention Reviews | Cochrane Methods [Internet]. [cited 2025 Sep 3]. Available from: https://methods.cochrane.org/methods-cochrane/random-effects-meta-analysis-methods-intervention-reviews?utm_source=chatgpt.com

[28] Bayesian random-effects meta-analysis of aggregate data on clinical events [Internet]. [cited 2025 Sep 3]. Available from: https://arxiv.org/html/2504.12214v1?utm_source=chatgpt.com 10.1002/sim.70508 PMC13044572 41923558

[29] Expert. PRISMA 2025 Update: 8 New Reporting Requirements Every Researcher Must Know [Internet]. Editverse. 2025 [cited 2025 Sep 3]. Available from: https://editverse.com/prisma-2025-update-8-new-reporting-requirements-every-researcher-must-know/

[30] Lakew S, Musema H, Shimeles T, Challinor J. Assessment of knowledge, accessibility and utilization of palliative care services among adult cancer patients at Tikur Anbesa Specialized Hospital, Addis Ababa, Ethiopia, 2014: a cross-sectional institution-based study. BMC Res Notes. 2015;8:657. doi: 10.1186/s13104-015-1790-0 26547548 PMC4636827

[31] Fetene D, Taylor L, Ferrell B, Deribe B, Abdella J, Aynalem A. Factors affecting need and utilization of palliative care services among Ethiopian women in an oncology department: a hospital-based cross-sectional study. Palliat Care Soc Pract. 2024;18:26323524241253625. doi: 10.1177/26323524241253625 38800039 PMC11127578

[32] Bunare A, Weldegebreal F, Habte S, Ketema I, Worku T. Utilization of rehabilitation services and associated factors among adults with cancer diagnoses at Hawassa comprehensive specialized hospital, Ethiopia. Patient Prefer Adherence. 2022;16:1571–80. doi: 10.2147/PPA.S370923 35791427 PMC9250770

[33] Kebebew T, Mosalo A, Mavhandu-Mudzusi AH. Hospital-based evaluation of palliative care among patients with advanced cervical cancer: a cross-sectional study. BMC Palliat Care. 2022;21(1):140. doi: 10.1186/s12904-022-01030-2 35918682 PMC9347143

[34] Worku T, Mengistu Z, Semahegn A, Tesfaye G. Rehabilitation for cancer patients at Black Lion hospital, Addis Ababa, Ethiopia: a cross-sectional study. BMC Palliat Care. 2017;16(1):53. doi: 10.1186/s12904-017-0235-7 29145841 PMC5693422

[35] Amare N, Gintamo B, Tukeni KN, Gebremichael EH, Abera EG. The prevalence of cancer patients requiring palliative care and its associated factors at St. Paul Hospital, Addis Ababa, Ethiopia: a cross-sectional study. Risk Manag Healthc Policy. 2023;16:1203–14. 37404288 10.2147/RMHP.S415532 PMC10315136

[36] Aynalem A, Abdella Muhammed J, Taylor L, Unverzagt S, Kroeber ES, Deribe B, et al. Utilization of palliative care and its associated factors among adult cancer patients in Hawassa University comprehensive specialized hospital oncology center, Hawassa, Ethiopia, 2021: a cross-sectional study. Curr Med Res Opin. 2023;39(6):911–7. doi: 10.1080/03007995.2023.2211915 37158021

[37] Mamo Y, Habte A, W/Giorgis N, Abreha A, Ayers N, Abathun E, et al. The evolution of hospice and palliative care in Ethiopia: From historic milestones to future directions. Ethiopian J Health Dev. 2020;34(4). Available from: https://www.ejhd.org/index.php/ejhd/article/view/3648

[38] Kaba M, de Fouw M, Deribe KS, Abathun E, Peters AAW, Beltman JJ. Palliative care needs and preferences of female patients and their caregivers in Ethiopia: a rapid program evaluation in Addis Ababa and Sidama zone. PLoS One. 2021;16(4):e0248738. doi: 10.1371/journal.pone.0248738 33886561 PMC8062072

[39] Kimani CW, Kioko UM, Ndinda C, Adebayo PW. Factors influencing progressive utilization of palliative care services among cancer patients in Kenya: the case of Nairobi Hospice. Int J Environ Res Public Health. 2023;20(19):6871. doi: 10.3390/ijerph20196871 37835141 PMC10572499

[40] Guma S, Namisango E, Ddungu H. Advance care planning and palliative care for patients with advanced cancer in Uganda. Zeitschrift für Evidenz, Fortbildung und Qualität im Gesundheitswesen. 2023;180:29–35. 37550121 10.1016/j.zefq.2023.07.002

[41] Getie A, Yilak G, Ayenew T, Amlak BT. Palliative care utilisation globally by cancer patients: systematic review and meta-analysis. BMJ Support Palliat Care. 2025;15(3):291–9. doi: 10.1136/spcare-2024-005000 40032593 PMC12128768

[42] Namukwaya E, Mwaka AD, Namisango E, Mwesiga MD, Downing J. Current State of Palliative care in Uganda. In: Silbermann M, editor. Palliative care for Chronic Cancer Patients in the Community: Global Approaches and Future Applications [Internet]. Cham: Springer International Publishing; 2021. pp. 267–78. [cited 2025 Apr 10]. Available from: doi: 10.1007/978-3-030-54526-0_24

[43] Lee H-TS, Yang C-L, Leu S-V, Hu W-Y. Cultural impediments to frank communication regarding end-of-life care between older nursing home residents and their family members in Taiwan: a qualitative study. BMC Nurs. 2022;21(1):357. doi: 10.1186/s12912-022-01143-2 36517826 PMC9749315

[44] Kulikowski JD, Namisango E, Rosa WE. Patients with advanced cancer in Uganda: Gender, social norms, and family relationship icebergs in the face of terminal illness. Palliat Support Care. 2025;23:e31. doi: 10.1017/S1478951524002189 39834191 PMC12145489

[45] Eleke C, Azuonwu G, Agu IS, Nnorom RM, Ogini AN, Eleke-Bempong E, et al. Knowledge of palliative care among professional nurses in south east Nigeria: A needs assessment for continuing education. Int J Afr Nurs Sci. 2020;13:100237. doi: 10.1016/j.ijans.2020.100237

[46] Urrizola A, Lundeby T, Hjermstad M, Deliens L, Caraceni A, Fallon M. The Role of Health Literacy and Care Pathways in the Provision of Palliative Care and Patient-Centred Care: A Discussion Paper. In: Hole T, Kvangarsnes M, Landstad BJ, Bårdsgjerde EK, Tippett-Spirtou SE, editors. Towards Sustainable Good Health and Well-being: The Role of Health Literacy. Cham: Springer Nature Switzerland; 2025. pp. 183–202. Available from: doi: 10.1007/978-3-031-61810-9_11

[47] Wong KTC, Chow AYM, Chan IKN. Effectiveness of educational programs on palliative and end-of-life care in promoting perceived competence among health and social care professionals. Am J Hosp Palliat Care. 2022;39(1):45–53. doi: 10.1177/10499091211038501 34409883

[48] Anago EK, McFeely C, Bayuo J, Suglo JN, Friesen LD. Experiences of adult informal caregivers in palliative care provision for persons with advanced cancer: a meta-synthesis. J Pain Symptom Manage. 2026;71(1):e40–68. doi: 10.1016/j.jpainsymman.2025.08.042 40907621

[49] Merriman A, Mwebesa E, Zirimenya L. Improving access to Palliative care for patients with cancer in Africa: 25 years of Hospice Africa [Internet]. 2019 [cited 2025 Sep 5]. Available from: http://ecancer.org/en/journal/article/946-improving-access-to-palliative-care-for-patients-with-cancer-in-africa-25-years-of-hospice-africa 10.3332/ecancer.2019.946 PMC6722241 31552119

[50] Harding R, Nair S, Ekstrand M. Multilevel model of stigma and barriers to cancer palliative care in India: a qualitative study. BMJ Open. 2019;9(3):e024248. doi: 10.1136/bmjopen-2018-024248 30837250 PMC6429952

[51] Obiezu-Umeh C, Ogbenna AA, Johnson LS, Caputo MT, Hirschhorn LR, Onyeka TC, et al. Barriers and facilitators to the implementation of palliative care services at five tertiary hospitals in Nigeria: a qualitative formative study. BMC Health Serv Res. 2025;25(1):970. doi: 10.1186/s12913-025-13138-1 40696352 PMC12285048

[52] Bassah N, Santos Salas A, Ambe NC, Ngah NE. Increasing access to palliative care in Cameroon: progress, gaps, and recommendations. Nurs Rep. 2024;14(4):3606–18. 39585154 10.3390/nursrep14040263 PMC11587453

[53] Sítima G, Galhardo-Branco C, Reis-Pina P. Equity of access to palliative care: a scoping review. Int J Equity Health. 2024;23(1):248. doi: 10.1186/s12939-024-02321-1 39581966 PMC11587758

[54] Cheboi SK, Nyawira DN, Ngolo PM, Ng’ang’a WS. Determinants of patient satisfaction in indigenous cancer palliative care services. Inquiry. 2023;60. doi: 10.1177/00469580231167733 PMC10108425 37057317

[55] Schelin MEC, Fürst CJ, Rasmussen BH, Hedman C. Increased patient satisfaction by integration of palliative care into geriatrics-a prospective cohort study. PLoS One. 2023;18(6):e0287550. doi: 10.1371/journal.pone.0287550 37347730 PMC10286968

[56] Sandsdalen T, Wilde-Larsson B, Grøndahl VA. Patients’ perceptions of the quality of palliative care and satisfaction - a cluster analysis. J Multidiscip Healthc. 2019;12:903–15. doi: 10.2147/JMDH.S220656 31806988 PMC6857653

[57] Dipio R, Acuda W, Namisango E, Nalubega-Mbowa MG. Prevalence and factors associated with depressive symptoms among family caregivers of palliative care patients at Hospice Africa Uganda. Palliat Support Care. 2022;20(3):375–82. doi: 10.1017/S1478951521000730 34154696

[58] Paterson LA, Maritz JE. Nurses’ experiences of the family’s role in end-of-life care. Health SA Gesondheid (Online). 2024;29:2565. doi: 10.4102/hsag.v29i0.2565 38841354 PMC11151341

