# Supplementary material for: Utilization of palliative care services and associated factors among cancer patients in Ethiopia: A systematic review and meta-analysis
Source: PLoS One. 2026 Mar 23;21(3):e0345299. doi: 10.1371/journal.pone.0345299 (PMC13008075; doi:10.1371/journal.pone.0345299)
Supplement: S1 File — (DOCX) [file pone.0345299.s001.docx]

| Section/topic | **#** | **Checklist item** | Reported on page # |
| --- | --- | --- | --- |
| **Tittle** | | | |
| **Tittle** | **1** | **Utilization of Palliative Care Services and Associated Factors among Cancer Patients in Ethiopia: A Systematic Review and Meta-Analysis** | **P1** |
| **Abstract** | | | |
| **Structured summary** | **2** | Introduction: Palliative care is vital for cancer management in low- and middle-income countries like Ethiopia, but underutilization leads to unmanaged symptoms and reduced patient quality of life, and fragmented studies hinder evidence-based planning and policy development. This systematic review and meta-analysis aim to synthesize existing literature to estimate the utilization rate of PC among cancer patients in Ethiopia and to identify key influencing factors.  Review Method and Data Sources: This study employed a systematic review and meta-analysis design to assess palliative care utilization and its influencing factors among cancer patients in Ethiopia, sourcing evidence from various electronic databases until April 07, 2025. The data was extracted from March 10-20 and analyzed from March 21-30, with report generation till April 07, 2025, using R software. Meta-analysis was performed using a random-effects model, with forest plots illustrating pooled prevalence and associated factors. Heterogeneity was assessed using the I² statistic, and study quality was evaluated using a validated tool.  Results: A total of nine cross-sectional studies involving 2,839 cancer patients were included. The pooled palliative care utilization rate was 40.2% (95% CI: 28.3%–52.0%). Palliative care utilization was significantly higher among patients with higher educational levels, males, those with better income, proximity to health facilities, and those reporting higher satisfaction with services. Institutional care dominated Palliative care delivery, primarily in urban hospitals, while rural and community-based services were scarce.  Conclusion: This review showed palliative care utilization rate was 40.2%. Palliative care utilization among cancer patients in Ethiopia remains limited. Addressing these gaps through decentralization of services, integration of Palliative care into primary healthcare, investment in workforce development, and policy reforms is essential for improving equity and outcomes in palliative care delivery. | **P2** |
| **Introduction** | | | |
| **Rationale** | **3** | While individual studies have attempted to investigate palliative care utilization and its determinants among cancer patients in Ethiopia, findings are fragmented and sometimes contradictory, thereby limiting the generalizability and applicability of the evidence. To date, there has been no comprehensive synthesis of these studies using meta-analytic techniques to provide pooled estimates and identify the most influential factors affecting palliative care utilization in the Ethiopian context. A systematic review and meta-analysis are therefore needed to fill this gap by summarizing existing knowledge, quantifying utilization rates, and identifying consistent predictors across various settings within the country. Such evidence is vital not only for national policy development but also for regional and international efforts to promote equitable access to palliative care for all. | **P5** |
| **Objectives** | **4** | This systematic review and meta-analysis aim to assess the utilization of palliative care (PC) services and the influencing factors among cancer patients in Ethiopia | **P5** |
| **METHODS** | | | |
| **Protocol and registration** | **5** | We registered for this study with the CRD420251027739 registration number. | **P7** |
| **Eligibility criteria** | **6** | Included in the review are published and unpublished observational and interventional studies reporting on PC utilization and its influencing factors among adult cancer patients in Ethiopia. Eligible studies must provide data on demographics, service accessibility, types of PC provided, and utilization outcomes. Peer-reviewed articles, theses, dissertations, and relevant gray literature written in English were considered without date restrictions | **P6** |
| **Information sources** | **7** | A systematic search was conducted in electronic databases including PubMed, Scopus, Web of Science, Google Scholar, African Journals Online (AJOL), and Ethiopian university repositories. Search terms included combinations of: “palliative care,” “PC utilization,” “cancer,” “oncology,” “Ethiopia,” and “associated factors.” Boolean operators and Medical Subject Headings (MeSH) terms were used where applicable. Reference lists of selected articles were also manually screened. No restrictions on publication year and language were applied. Duplicate records were removed, and two independent reviewers screened the titles, abstracts, and full texts to ensure the inclusion of eligible studies. Discrepancies were resolved through discussion or consultation with each other. The database was searched for every article published on palliative care utilization among cancer patients till March 10, 2025, and continued to update until we sent it for publication. The data was extracted from March 10-20 and later analyzed from March 21-30, and the report generation till April 07, 2025 | **P6-7** |
| **Search** | **8** | Listed in appendix as table 01 | **P1-5** |
| **Study selection** | **9** | A total of 56 records were identified across databases: PubMed (14), CINAHL (04), Scopus (10), Web of Science (12), and Google Scholar (16). After removing duplicates and applying inclusion criteria, 16 full-text articles were assessed. Nine studies met all inclusion criteria and were included in the final analysis. Screening and data extraction were conducted independently by two reviewers, with discrepancies resolved through discussion (Figure 1). | **P7** |
| **Data collection process** | **10** | Based on the Joanna Briggs Institute methodology principles, a data extraction template was created. First author, publication year, nation, study goal, underlying concept, study design, locations, participants, sample size, sampling technique, data collection techniques, important findings, suggestions, and study limitations were all taken out of each. Differences were settled by discussion between the two reviewers who separately reviewed and extracted data from the full-text versions of the included publications. | **P7** |
| **Data items** | **11** | For analysis, data were taken out of the included studies and exported to Excel. Narrative descriptive statistics (frequency, percentages, mean, and range) were utilized to assess quantitative findings. The PRISMA-Sco checklist served as our guide for reporting the findings. Tables, figures, and a descriptive manner are used to present the results. The table presents all of the extracted data in a manner that addresses the review question. | **P8-9** |
| **Risk of bias in individual studies** | **12** | The methodological quality of the included studies was evaluated using the Joanna Briggs Institute (JBI) Critical Appraisal Checklist for Analytical Cross-Sectional Studies. Each study was independently assessed by two reviewers across domains such as sample selection, outcome measurement, control of confounding variables, and statistical analysis. Studies scoring 7–8 “Yes” responses were considered low risk of bias, 4–6 as moderate risk, and 0–3 as high risk. Only low- and moderate-risk studies were included in the final synthesis (Table 2). | **P9** |
| **Summary measures** | **13** | In this systematic review, PC utilization among nurses was assessed descriptively using narrative synthesis, without conducting a meta-analysis. The primary measures extracted from the studies included the prevalence of personal computer usage, purposes for usage (such as data entry, report writing, and communication), and associated factors. | **P9** |
| **Synthesis of results** | **14** | Data analysis was performed using R software. Descriptive statistics (frequencies, percentages, means, and standard deviations) were used to summarize study characteristics. A meta-analysis using a random-effects model pooled estimates for PC utilization and significant predictors. Heterogeneity was evaluated using the I² statistic (I² > 50% indicating substantial heterogeneity). Egger’s test and funnel plots assessed publication bias. Sensitivity analyses were conducted by excluding studies with high bias or small sample sizes. All results were reported with 95% confidence intervals, and statistical significance was set at p < 0.05. | **P9** |
| Risk of bias across studies | **15** | The methodological quality of the included studies was evaluated using the Joanna Briggs Institute (JBI) Critical Appraisal Checklist for Analytical Cross-Sectional Studies. Each study was independently assessed by two reviewers across domains such as sample selection, outcome measurement, control of confounding variables, and statistical analysis. Studies scoring 7–8 “Yes” responses were considered low risk of bias, 4–6 as moderate risk, and 0–3 as high risk. Only low- and moderate-risk studies were included in the final synthesis (Table 2). | **P9** |
| Additional analyses | **16** | Pooled utilization rate, sensitivity analysis, and pooled of associated factors was on the result section | **P10-15** |
| **RESULTS** | | | |
| **Study selection** | **17** | The included studies were all cross-sectional in design and conducted within various institutional settings across Ethiopia, primarily in public hospitals and specialized oncology centers (see table 1). | **P10** |
| **+Study characteristics** | **18** | All included article (9 in numbers) were Cleary characterized in table 1 | **P 1-7** |
| **Risk of bias within studies** | **19** | For each article it was stated as limitation in the table one | **P 1-7** |
| **Results of individual studies** | **20** | All included article (9 in numbers) were Cleary characterized in table 1 | P 1-7 |
| **Synthesis of results** | **21** | All included article (9 in numbers) were Cleary characterized in table 1 | P 1-7 |
| **Risk of bias across studies** | **22** | For each article it was stated as limitation in the table one | **P 1-7** |
| **Additional analysis** | **23** | Pooled utilization rate, sensitivity analysis, and pooled of associated factors was on the result section | P10-15 |
| DISCUSSION | | | |
| **Summary of evidence** | **24** | This systematic review and meta-analysis highlight the moderate yet insufficient utilization of palliative care services among cancer patients in Ethiopia, with a pooled prevalence of 40.2%. Despite growing recognition of its importance, palliative care remains largely limited to institutional settings in urban areas, leaving rural and underserved populations with minimal access. The findings reveal critical disparities influenced by socio-demographic factors such as education, income, gender, and proximity to healthcare facilities, as well as systemic barriers including lack of trained personnel, inadequate community-based services, and weak integration into the national health system. While progress has been made in expanding service components such as pain and symptom management, other essential aspects like psychosocial and spiritual support remain underdeveloped. Addressing these challenges will require a concerted effort to decentralize palliative care delivery, improve health literacy, invest in workforce development, and implement policy reforms aimed at achieving equitable, patient-centered care across the country. | **P 20** |
| **Limitations** | **25** | All included studies employed cross-sectional designs, limiting the ability to establish causal relationships. Additionally, variations in outcome measures across studies hindered comparability, and the scarcity of data from rural and community-based settings may have led to an underrepresentation of the most underserved populations and included in the table 1 as well | **P 20 and P1-7** |
| **Conclusions** | **26** | This review showed palliative care utilization rate was 40.2%. Palliative care utilization among cancer patients in Ethiopia remains limited. Addressing these gaps through decentralization of services, integration of Palliative care into primary healthcare, investment in workforce development, and policy reforms is essential for improving equity and outcomes in palliative care delivery. | **P 20** |
| FUNDING | | | |
| **FUNDING** | **27** | There were no grants awarded to the evaluated study by public, private, or nonprofit funding organizations. | **P 21** |
