# Supplementary material for: Utilization of palliative care services and associated factors among cancer patients in Ethiopia: A systematic review and meta-analysis
Source: PLoS One. 2026 Mar 23;21(3):e0345299. doi: 10.1371/journal.pone.0345299 (PMC13008075; doi:10.1371/journal.pone.0345299)
Supplement: S2 File — (DOCX) [file pone.0345299.s002.docx]

**Supporting file 2.** Search Strategy and Retrieval Summary for the systematic review and meta-analysis on Palliative care among cancer patients in Ethiopia, 2025.

| **Database** | **Search Strategy (keywords/MeSH terms)** | **Records Retrieved** | **After Duplicates Removed** | **Full-texts Assessed** | **Studies Included** |
| --- | --- | --- | --- | --- | --- |
| PubMed | (“palliative care”[MeSH] OR “palliative care utilization” OR “end-of-life care”) AND (“cancer” OR “oncology”) AND “Ethiopia” | 24 | 18 | 5 | 3 |
| Scopus | (TITLE-ABS-KEY (“palliative care” OR “end-of-life care”) AND TITLE-ABS-KEY (“cancer”) AND TITLE-ABS-KEY (“Ethiopia”)) | 14 | 10 | 4 | 2 |
| Web of Science | (“palliative care” OR “end-of-life care”) AND (“cancer”) AND (“Ethiopia”) | 12 | 9 | 3 | 1 |
| CINAHL | (MH “Palliative Care” OR “end-of-life care”) AND (MH “Cancer” OR “Oncology”) AND Ethiopia | 16 | 11 | 2 | 1 |
| AJOL (African Journals Online) | “Palliative care” AND “cancer” AND “Ethiopia” | 9 | 6 | 1 | 1 |
| Google Scholar | Allintitle: “palliative care” AND “cancer” AND “Ethiopia” | 16 | 12 | 1 | 1 |
| Ethiopian University Repositories | “palliative care utilization” AND “cancer” AND “Ethiopia” | 1 | 1 | 0 | 0 |
| **Total** | 82 | 65 | 16 | 9 |  |
