# Supplementary material for: Utilization of palliative care services and associated factors among cancer patients in Ethiopia: A systematic review and meta-analysis
Source: PLoS One. 2026 Mar 23;21(3):e0345299. doi: 10.1371/journal.pone.0345299 (PMC13008075; doi:10.1371/journal.pone.0345299)
Supplement: Attachment — Submitted filename: Peer Review PONE - D - 25 - 41618R1.pdf [file pone.0345299.s005.pdf]

Introduction

1. Remove and in the line “leading to late stage cancer diagnoses AND”. Replace with comma
2. Recommend not abbreviating palliative care, given this is the highlight of the entire paper
3. Inconsistent use of the acronyms. LMIC was already defined then spelled out in the 1st line of the 4th paragraph.
4. “Estimates suggesting only a fraction receive it” is repetitive of the point made palliative care remains insufficient. Give “the fraction” numerically if you are going to use the word “fraction” in that regard
5. The 3rd paragraph lists factors why palliative care is underutilized globally then the 4th paragraph lists factors why it is underutilized in LMIC. Condense and revise
6. The 5th paragraph does not add anything to the introduction. Consider condensing with the above 2 paragraphs as the reader has already understood the lack of palliative care services
7. 6th paragraph too small and lacking necessary information to stand alone as a paragraph.
8. Same for the 6th paragraph beginning with “There is a growing..”. Condense with above and revise
9. “...to at least 50% of public facilities by 2020”. 2020 was 6 years ago. What is the relevance of this now?

Results

1. Helpful to add a table showing visually the factors looked at and the Odds ratios with CI and the ranges
2. Given that distance was discussed as well, consider adding a geographical representation to help aid the reader visualize the distances the patients had to maneuver as well
3. Funnel and Egger are not reliable with < 10 studies. Ensure adding that even though absence of bias was not detected does not mean it was absent
4. Effect sizes and CI are inconsistent in different sections
5. Bayesian analysis had no results
6. No comment noted on multiple comparisons

Discussion

1. Advise not putting “recommendations” such as in paragraph 2 so early on in the discussion. Consider condensing at the end
2. Helpful to put the value seen after “markedly low” in 1st paragraph
3. Change in text formatting font noted throughout the discussion. Please correct
4. Why are there comparisons to Taiwan? Would the surrounding countries of Ethiopia be better? Odd to single out a south east asian country at random
5. “Good knowledge” is not appropriate english

The discussion has notable flaws and each paragraph ends with vague recommendations that don't really hone in actionable tasks to do. Seems as if it lists out general tasks to help which can be discerned without this study as well. The study really condensed 9 articles however most of the answers to the actual questions remain in the details that are postulated for the research question. I would truly recommend re doing the discussion and either condensing the recommendations to help develop the system at the end rather than scatter throughout or focus on the key important points.
