# Supplementary material for: Utilization of palliative care services and associated factors among cancer patients in Ethiopia: A systematic review and meta-analysis
Source: PLoS One. 2026 Mar 23;21(3):e0345299. doi: 10.1371/journal.pone.0345299 (PMC13008075; doi:10.1371/journal.pone.0345299)
Supplement: Attachment — Submitted filename: Peer Review PONE - D - 25 - 41618 R2.pdf [file pone.0345299.s007.pdf]

Introduction

1. Remove “with” before “70% of these deaths..”
2. Excessive commas noted in the line “Despite its proven…”
3. Revise line “these challenges are particularly”. Excessively long and unnecessary commas again
4. Revise paragraph 4. Excessive commas. Please avoid consistent listing of challenges or problems. Consider grouping and revising to allow easier flow. At a certain point with too many listings the importance of the factors lose importance to the reader.
5. Should “international partners” be a common I?
6. Revise line “Over the past two decades”. Same problem as above
7. Should “National health sector” be a common N?
8. What gap is highlighted? Helpful

Methods

1. Excessive “and” in methods section of “ Aim of the study”
2. Palliative care is written in common letters and at different times with a Capital P. Please ensure homogeneity
3. Why is “search strategy” in a different font?
4. Several parts ot the method section has different fonts utilized and irregular spacing with the sub heading with the paragraph
5. Why is Funnel plot and egger’s mentioned here again?

Results

1. Please revise paragraph 1 in the Palliative care service utilization rate section. Too many percentages listed one after the other. A range may be better
2. Inconsistent font noted in paragraphs before discussion heading

Discussion

1. Irregular fonts noted throughout the discussion

Additional Comments

1. Ensure its clearly communicated after the pool prevalence of 42% that given the high heterogeneity the estimate should be interpreted as a descriptive aggregate rather than definitive
2. P = 0.00 is not significant to mention as it holds no true value. Reconsider how you are presenting this aspect
3. Esnure the predictors are correctly identified and explained as associations of palliative care utilization and interpreted correlationally
4. More interpretation about why the AOR for Gender being > 5 needs to be fleshed out
5.
