# Supplementary material for: Utilization of palliative care services and associated factors among cancer patients in Ethiopia: A systematic review and meta-analysis
Source: PLoS One. 2026 Mar 23;21(3):e0345299. doi: 10.1371/journal.pone.0345299 (PMC13008075; doi:10.1371/journal.pone.0345299)
Supplement: Attachment — Submitted filename: Response for reviewers.docx [file pone.0345299.s006.docx]

**Response for the reviewers**

**Response to Reviewer #1 Comments**

We sincerely thank Reviewer #1 for the thoughtful and constructive feedback provided on our manuscript titled “Palliative Care Service Utilization among Cancer Patients in Ethiopia.” We greatly appreciate the recognition of our work’s relevance and methodological rigor. Below we provide a point-by-point response to each of the reviewer’s comments, along with the revisions made in the manuscript.

**1. Remove the year 2025 from the title**

Response: We agree with the reviewer. The year has been removed from the title.

Change made: Title now reads: “Palliative Care Service Utilization among Cancer Patients in Ethiopia: A Systematic Review and Meta-Analysis.”

**2. Abstract – include pooled effect size for associated factors**

Response: We have revised the results section of the abstract to include pooled effect sizes of significant associated factors (e.g., education, gender, income, access, satisfaction). Change made: Abstract now reports, for example: “Educational attainment (pooled AOR = 2.57; 95% CI: 1.42–3.75), male gender (AOR=5.58; 95% CI: 3.01–10.33), higher income (AOR = AOR = 26.9, 95% CI: 12.3–59), proximity to health facilities (AOR = 2.24; 95% CI: 1.52–3.29).), and satisfaction with care (AOR = 1.96; 95% CI: 1.30–2.95) ,and family and social support (AOR = 12.18; 95% CI: 1.29–3.68) were significantly associated with palliative care utilization.”

**3. Search strategy – present full algorithm and retrieval numbers as a table**

Response: We have prepared a supplementary table detailing the complete search strategies (keywords, Boolean operators, MeSH terms) for each database, along with the number of records retrieved before and after duplicate removal.

Change made: Added as “Table 1: Search Strategy and Retrieval Summary.”

**4. Methodology section lacks citations**

Response: Additional references have been inserted to support methods used, including PRISMA, Joanna Briggs Institute checklist for quality appraisal, and standard texts on random-effects meta-analysis and heterogeneity.

Change made: Appropriate citations added throughout methodology.

**5. Heterogeneity results and subgroup analysis**

Response: We clarified heterogeneity results within the forest plots by presenting I² values directly on figures. In addition, we conducted subgroup analysis stratified by study region (Addis Ababa vs. outside Addis Ababa) to explore potential sources of heterogeneity. Results are now presented in the results section

Change made: Figures updated with I² values; new figures showing subgroup pooled prevalence included.

**6. Q statistic results**

Response: We now explicitly report Cochran’s Q statistic outputs in the results section and display them alongside I² and τ² estimates.

**7. Discussion – comparison with African countries**

Response: We revised the discussion to include comparison with studies from other African countries (e.g., Uganda, Kenya, South Africa, Nigeria), in addition to the previously cited global literature.

**8. Limitation – hospital-based studies not generalizable**

Response: We agree and have added this limitation to the limitations section: “As most included studies were hospital-based and concentrated in urban referral centers, the findings may not fully reflect palliative care realities in rural and community settings across Ethiopia.”

Change made: Added to limitations paragraph

**9. Citations, typos, and grammar**

Response: The entire manuscript has been carefully proofread to correct typographical and grammatical errors, and to ensure reference formatting consistency.

We are grateful for the reviewer’s insightful comments, which have substantially improved the clarity, methodological transparency, and contextual relevance of our manuscript. We believe these revisions have strengthened the overall quality and rigor of our work.

**Response for Reviewer Two**

We sincerely thank Reviewer #2 for the careful and constructive review of our manuscript titled “Palliative Care Service Utilization among Cancer Patients in Ethiopia: A Systematic Review and Meta-Analysis, 2025.” We greatly appreciate the insightful comments and suggestions, which have helped us strengthen the clarity, scientific rigor, and overall quality of the paper. We have carefully revised the manuscript in response to each point, as detailed below. All changes have been incorporated into the appropriate sections of the manuscript, and specific additions or modifications are highlighted in our responses.

**General Comments**

1. The paper is well written and organized, though some modifications are advised.
   **Response:** Thank you. We have revised the manuscript to improve clarity, flow, and readability.
2. Attention is needed to improve sentence synthesis and grammar throughout the manuscript.
   **Response:** We carefully proofread the manuscript and corrected grammar and sentence structure issues throughout.

**Abstract**

1. Do not use abbreviations in the abstract.
   **Response:** We replaced abbreviations (e.g., PC → “palliative care”) with full terms.
2. Specify publication year range boundaries of included studies.
   **Response:** We now state: “Studies published between 2015 and 2024 were included…”
3. Name the tool used for quality appraisal.
   **Response:** We added: “The Joanna Briggs Institute (JBI) Critical Appraisal Checklist was used.”
4. Include pooled odds ratios with CIs in results summary.
   **Response:** We added pooled AORs (e.g., education, gender, income, access, satisfaction).
5. Conclusion reads like results—emphasize implications and recommendations.
   **Response:** We revised the conclusion to highlight implications for policy and practice rather than re-listing results.

**Introduction**

Needs to be more comprehensive: what has been accomplished in Ethiopia, current plan, obstacles, SGD links.

**Response:** We expanded the introduction to include:

- Ethiopia’s Ministry of Health initiatives for integrating palliative care.
- Gaps such as lack of workforce, poor infrastructure, limited rural coverage.
- Links to Sustainable Development Goal (SDG 3: Ensure healthy lives…).

**Change:** Introduction, last three paragraphs.

**Methods**

1. Research questions not specific.
   **Response:** Revised to:
   - “What is the pooled prevalence of palliative care utilization among cancer patients in Ethiopia?”
   - “What are the key factors associated with utilization?”
     **Change:** Methods, Research Questions subsection.
2. Why restrict papers based on factors?

**Response to Reviewer:** We appreciate this important comment. We did not exclude papers solely based on whether they reported factors; rather, our primary objective was to estimate both the pooled prevalence of palliative care utilization and to identify associated factors. Therefore, we required that included studies provide either prevalence data or factors influencing utilization to allow for meaningful synthesis. Studies that did not report on either outcome (e.g., opinion pieces, reviews, purely descriptive articles without utilization data) were excluded because they could not contribute to the quantitative or qualitative objectives of this meta-analysis. However, both prevalence-only and factor-only studies were included where appropriate.

3. Why exclude non-Ethiopian patients or non-human studies?

Response to Reviewer: Thank you for raising this point. Non-human studies were excluded because our review focused exclusively on human health outcomes and service utilization, which cannot be inferred from laboratory or animal studies. Similarly, we excluded studies that reported on non-Ethiopian populations, even if they were treated in Ethiopian hospitals, to maintain national representativeness and ensure the pooled estimates reflected the experiences of patients residing within the Ethiopian healthcare system. Including non-Ethiopian patients could have introduced bias, given potential differences in health-seeking behavior, cultural context, and eligibility for local health services.

**Results**

1. Some studies did not report prevalence with odds ratios—how managed?

Response to Reviewer**:** Thank you for this valuable observation. In cases where studies did not report odds ratios directly, we extracted raw frequency data (when available) to calculate crude odds ratios with corresponding 95% confidence intervals. For studies where the necessary raw data were not provided, we did not attempt to impute missing values; instead, these findings were narratively synthesized and presented in the results section to avoid data distortion. This approach allowed us to include all eligible studies while maintaining methodological transparency.

2. All papers have same measurement—why difficult to pool?

Response to Reviewer: We appreciate this important comment. Although all included studies assessed palliative care utilization, the operational definitions and measurement approaches varied. Some studies defined utilization as the proportion of patients who had ever received palliative care services, while others measured expressed need, attendance at specific service components (e.g., symptom control, rehabilitation), or satisfaction with services as a proxy indicator. Additionally, data collection tools differed—ranging from structured questionnaires to medical record reviews—introducing methodological heterogeneity. Because of these variations, pooling results directly was challenging; therefore, we carefully standardized measures when possible and explained methodological differences when standardization was not feasible.

3. Are Addis Ababa and Hawassa enough to show regional variation?
   **Response:** Data for subgroup analysis were available only from Addis Ababa and Hawassa. We acknowledge that these cities do not represent all regions of Ethiopia, and regional variation may not be fully captured. Consequently, interpretations of regional differences should be made cautiously. We add in the limitation as This study includes data only from Addis Ababa and Hawassa, which may not represent all regions of Ethiopia. As a result, the findings might not fully reflect regional variations in the studied outcomes.
4. Why include factors like age/side effects from one study?

Response to Reviewer: We included factors such as age and treatment side effects even though they were reported in only one study each. We did this to provide a comprehensive overview of all reported factors associated with palliative care utilization in Ethiopia. We have clarified in the manuscript that these findings are based on individual studies and should be interpreted cautiously. This approach ensures transparency while highlighting gaps in the current evidence base.

5. Pooling and explanation of factors not clear.

**Response:** We reorganized results with clear subtitles (Education, Gender, Income, Access, Satisfaction, etc.), each with citations and pooled AORs where available.
Only factors reported in ≥2 studies were pooled quantitatively; factors reported in a single study (e.g., ADLs, knowledge, employment) were summarized narratively. This approach ensures a comprehensive synthesis while maintaining transparency about the strength of the evidence.

**Discussion**

1. Avoid too much information outside study objectives.
   **Response:** We removed unrelated global details and kept discussion focused on Ethiopian context and comparisons.
2. Remove redundancy of results.
   **Response:** We deleted repeated numerical results and emphasized interpretation.

**Conclusion & Recommendation**

1. Reorganize to be specific to findings.
   **Response:** Conclusion now highlights:
   - Utilization is only 40.2%
   - Inequities by gender, education, income, and geography
   - Need for decentralized services, training, and integration into primary care
     **Change:** Conclusion section rewritten.

Once again, we are grateful for the reviewer’s thoughtful feedback, which has significantly improved the quality of our manuscript. We believe that the revisions have addressed all concerns and enhanced the scientific soundness, clarity, and contribution of the study. We respectfully resubmit the revised manuscript for your further consideration and hope it will now be suitable for publication.
