# Supplementary material for: Utilization of palliative care services and associated factors among cancer patients in Ethiopia: A systematic review and meta-analysis
Source: PLoS One. 2026 Mar 23;21(3):e0345299. doi: 10.1371/journal.pone.0345299 (PMC13008075; doi:10.1371/journal.pone.0345299)
Supplement: Attachment — Submitted filename: review.docx [file pone.0345299.s004.docx]

How do you measure outcome

Certainty of evidence is a part of the PRISMA checklist, and is not currently part of the methodology. I suggest using the recently published Core GRADE which is a streamlined tool to assess the overall certainty post-meta-analysis.

The results are interpreted as valid, but all of the meta-analysis had extremely high heterogeneity as can be seen in the forest plots even with subgroup. As a consequence, these results are not valid for all. This should be communicated to readers so that they can interpret the findings accurately. Additionally, it would be beneficial to discuss what the sources of the heterogeneity may be.
