# Supplementary material for: Utilization of palliative care services and associated factors among cancer patients in Ethiopia: A systematic review and meta-analysis
Source: PLoS One. 2026 Mar 23;21(3):e0345299. doi: 10.1371/journal.pone.0345299 (PMC13008075; doi:10.1371/journal.pone.0345299)
Supplement: Attachment — Submitted filename: Author Response to Reviewers.docx [file pone.0345299.s008.docx]

**Author Response to Reviewers**

Author Response to Reviewer #1

We sincerely thank the reviewer for the careful re-review of our manuscript entitled “Palliative Care Service Utilization among Cancer Patients in Ethiopia: A Systematic Review and Meta-Analysis.” We appreciate the reviewer’s positive assessment of the improvements made and the constructive suggestions provided. We have addressed all remaining concerns in detail, as outlined below.

Comment 1- *“In the abstract to report the associated factors meta-analysis, the author should write only the pooled adjusted odds ratio (AOR) since this is the secondary analysis / pooling. If the result cannot pooled it can be summarized in the discussion part instead of repeating the results in the primary study.”*

Author Response: We thank the reviewer for this important methodological clarification. We fully agree that, as a secondary analysis, the abstract should report only pooled adjusted effect estimates derived from the meta-analysis, rather than repeating individual primary study results.

Accordingly, we have revised the Results section of the abstract to:

- Report only pooled adjusted odds ratios (AORs) with 95% confidence intervals for factors where quantitative pooling was feasible.
- Remove references to individual study-specific AORs that were not pooled.
- Exclude factors that could not be pooled quantitatively from the abstract and instead synthesize and interpret these findings narratively in the Discussion section, as recommended.

This revision ensures methodological rigor, avoids redundancy with primary studies, and aligns the abstract strictly with the results of the meta-analytic pooling.

Comment 2-*“The manuscript still have typo errors please see the result section of the abstract.”*

Author Response: We appreciate the reviewer for highlighting this issue. A thorough line-by-line proofreading of the entire manuscript has been conducted, with particular attention to the abstract and Results section.

Specifically:

- Typographical errors, duplicated words, punctuation issues, and formatting inconsistencies in the abstract were corrected.
- Redundant expressions and grammatical inaccuracies in numerical reporting (e.g., duplicated “AOR,” misplaced parentheses, and spacing issues) were carefully revised.
- Consistency in terminology (e.g., “palliative care,” “PC,” capitalization, and statistical notation) has been ensured throughout the manuscript.

These corrections have substantially improved the clarity, precision, and readability of the Results section and the manuscript as a whole.

**Comment 3-***“Similarly the manuscript should be clean.”*

**Author Response:** We fully acknowledge this comment and have taken comprehensive steps to ensure the manuscript is **clean, polished, and publication-ready**.

The following actions were undertaken:

- Full **language editing and grammatical revision** across all sections of the manuscript.
- Standardization of headings, subheadings, tables, figures, abbreviations, and statistical reporting.
- Removal of duplicated sentences, inconsistent phrasing, and minor stylistic errors.
- Harmonization of tense usage and academic tone throughout the text.
- Final formatting review to ensure consistency with journal expectations.

As a result, the revised manuscript now reflects a clear, coherent, and professionally edited scholarly work.

We are grateful to Reviewer #1 for the thoughtful and constructive feedback, which has significantly strengthened the methodological clarity, presentation, and overall quality of the manuscript. We believe that all concerns have now been fully addressed, and we respectfully submit the revised version for final consideration.

**Author Response to Reviewer #3**

We sincerely thank Reviewer #3 for the insightful and constructive comments. These suggestions have substantially improved the methodological rigor, transparency, and interpretability of our systematic review and meta-analysis. Below, we respond to each comment in detail and describe the corresponding revisions made to the manuscript.

Comment 1: The title should be modified to describe the objective.

Author Response: We agree with the reviewer that the title should more clearly reflect the study objectives, including both the estimation of utilization and the evaluation of associated factors.

Revision made: The title has been revised to explicitly reflect the objectives of estimating utilization and examining associated factors.

Original title: *Palliative Care Service Utilization among Cancer Patients in Ethiopia: A Systematic Review and Meta-Analysis*

Revised title: Utilization of Palliative Care Services and Associated Factors among Cancer Patients in Ethiopia: A Systematic Review and Meta-Analysis

Comment 2:

Publication bias should be tested using a Doi plot since this is a prevalence study.

Revised Response: We thank the reviewer for this important methodological recommendation. We carefully explored the use of the Doi plot and the Luis Furuya-Kanamori (LFK) index, which have been proposed as alternatives to funnel plots for meta-analyses of proportions and prevalence studies. We attempted to generate the Doi plot using both R and Stata by searching available packages and commands; however, we were unable to obtain a valid implementation suitable for our dataset.

Given this limitation, we assessed small-study effects and potential asymmetry using a Galbraith (radial) plot, which is commonly used in meta-analysis to evaluate heterogeneity and detect outlying or influential studies. The Galbraith plot did not indicate marked asymmetry or influential outliers, suggesting no substantial evidence of publication bias.

We have revised the manuscript accordingly and clearly described this methodological decision and its limitations.

Revisions made:

- Publication bias assessment was conducted using the Galbraith (radial) plot
- Attempts to apply the Doi plot were acknowledged, with justification for its non-application
- Funnel plot and Egger’s test were removed due to their limited suitability for prevalence data
- Methods and Results sections were updated accordingly

We inserted text (Methods – Statistical Analysis):“Publication bias and small-study effects were explored using the Galbraith (radial) plot. Although alternative approaches such as the Doi plot and LFK index have been recommended for prevalence meta-analyses, a valid implementation could not be obtained using available R for the current dataset.”

We Inserted text (Results – Publication Bias):“The Galbraith plot did not reveal marked asymmetry or influential outliers, suggesting no strong evidence of publication bias among the included studies.”

Sections revised:

- Methods → Statistical Analysis
- Results → Publication bias subsection
- Figure updated accordingly

Comment 3: Meta-analysis of predictors should include all studies, not only those that already found significant associations.

Author Response: We fully agree. The reviewer correctly notes that the objective of meta-analysis is to determine whether predictors remain significant after pooling all available evidence, not only those studies that reported significant associations.

Revision made:

- We revised the analytic approach for associated factors
- All studies reporting relevant predictors (regardless of statistical significance) were included in pooled analyses where sufficient data were available
- Forest plots (diamond plots) now reflect pooled estimates across all eligible studies
- Where pooling was not statistically or methodologically feasible, findings are clearly described as narrative synthesis

We Inserted clarification (Results – Factors section):“Predictors reported by fewer than two studies with comparable effect measures were synthesized narratively and interpreted cautiously.”

Comment 4: How is the outcome measured?

Response:
We thank the reviewer for highlighting the need for clearer outcome definition. We have now explicitly clarified how palliative care utilization was operationalized and measured across included studies.

Revision made: A dedicated clarification has been added describing outcome measurement approaches and their variability.

We Inserted text (Results – Palliative Care Service Measures):“Palliative care utilization was measured variably across studies, including receipt of at least one palliative care service, referral to a palliative care unit, self-reported utilization, or classification based on structured questionnaire scores. Although this variability reflects real-world practice, it contributes to between-study heterogeneity.”

Comment 5: Certainty of evidence (GRADE) is missing; recommend using Core GRADE.

Author Response: We appreciate this important suggestion. In response, we have incorporated Core GRADE, a streamlined and recently recommended approach, to assess the certainty of evidence for primary outcomes after meta-analysis.

Revision made:

- Added a Certainty of Evidence Assessment subsection
- Applied Core GRADE to pooled prevalence and major predictors
- Certainty ratings (low / very low) are now transparently reported and justified

We Inserted text (Methods – Certainty of Evidence):“The certainty of evidence was assessed using the Core GRADE approach, which provides a streamlined evaluation of overall certainty following meta-analysis, particularly suitable for observational studies.”

We Inserted text (Results – Certainty of Evidence): “Using the Core GRADE framework, the certainty of evidence for pooled palliative care utilization and associated factors was rated as low to very low, primarily due to high heterogeneity, cross-sectional study designs, and inconsistency across studies.”

Comment 6: Extremely high heterogeneity makes results not valid for all; this must be communicated and explained.

Author Response: We strongly agree. We have now explicitly acknowledged that the high heterogeneity limits the generalizability of pooled estimates and have expanded the discussion on potential sources of heterogeneity.

We Inserted text (Results): “All pooled analyses demonstrated substantial heterogeneity (I² > 90%), indicating considerable variability across studies. Consequently, pooled estimates should be interpreted as indicative rather than definitive national values.”

We Inserted text (Discussion – Heterogeneity):“The high heterogeneity likely arises from differences in outcome definitions, institutional capacity, urban–rural disparities, study populations, and availability of palliative care services. These findings underscore that utilization patterns are highly context-specific rather than uniform across Ethiopia.”

We sincerely thank Reviewer 3 for their careful and constructive comments, which have greatly contributed to improving the clarity, consistency, and scientific rigor of our manuscript. We have carefully addressed each point raised, including revisions to the results and discussion, consistent reporting of effect sizes and confidence intervals, and consolidation of recommendations. We believe that these changes have strengthened the manuscript and we greatly appreciate the reviewer’s thoughtful guidance.

**Author responses for Reviewer 4**

We sincerely thank Reviewer 4 for their thoughtful and constructive feedback on our manuscript. We greatly appreciate the detailed suggestions regarding the presentation of results, consistency of effect sizes, interpretation of findings, discussion structure, and the consolidation of recommendations. In response, we have carefully revised the manuscript to improve clarity, ensure consistency across results and discussion, focus on interpreting findings before providing recommendations, and consolidate actionable recommendations into a single section at the end of the discussion and conclusion.

**Point-by-Point Response to Reviewer #4**

INTRODUCTION

#1. “leading to late stage cancer diagnoses AND”

- Revision action:
   Remove “and” and replace with a comma.

Corrected sentence: “…leading to late-stage cancer diagnoses, resulting in higher mortality rates compared with high-income countries.”

#2. Do not abbreviate palliative care

Issue: You alternate between *palliative care*, *PC*, and *Palliative care*.

Revision action:

- We use “Palliative care” in full throughout (recommended for a concept central to the paper). Here in the first draft, I wrote as full Palliative care and after comments from the reviewer I change to PC after I defined first now, I change to full Palliative care currently thorough the paper.

3. Inconsistent use of LMIC

Issue: LMIC is defined, then later spelled out again.
Revision action:

- We define once at first mention.
- We use LMICs consistently thereafter.

4. “only a fraction receive it” is vague and repetitive

- Issue: Repeats “insufficient” without adding information.
  Revision action:
- We replace with a numerical estimate.

Revised sentence:-“Globally, it is estimated that fewer than 15% of individuals who need palliative care actually receive it.”(This aligns with Lancet Commission estimates and strengthens rigor.)

5–6. Redundant paragraphs on global vs LMIC barriers

Issue: Paragraphs beginning *“Sub-Saharan Africa…”* and *“There is a growing…”* are too thin.
Revision action: we merge with adjacent paragraphs and expand with specific context (Ethiopia-focused).

7–8. Very short paragraphs

Issue: Paragraphs 3–5 repeat similar barriers.
Revision action:

- We condense into ONE coherent paragraph, structured as: global brief LMIC-specific intensifiers (focus) and Ethiopia as a case example. This improves flow and reduces reviewer fatigue.

9. “50% of public facilities by 2020”

Issue: Policy target is outdated.
Revision action:

- Reframe historically and critically.

Revised framing: -Ethiopia has prioritized strengthening palliative care as an essential component of Universal Health Coverage, aligning with Sustainable Development Goal 3 and national health sector strategies. Although the country set a target to integrate palliative care and pain management services into at least 50% of public health facilities by 2020, evidence from more recent studies indicates that service availability and utilization remain limited, particularly outside major urban centers (19). This gap highlights the need for accelerated decentralization of palliative care services, strengthened workforce capacity through pre-service and in-service training, expansion of community- and home-based delivery models, and better integration of palliative care into primary healthcare systems to advance national UHC and SDG commitments (19,22,23).” This shows awareness and avoids appearing out of date.

RESULTS

1. Add a summary table of factors

Revision action:

- We add a single table summarizing: the factors associated with palliative care utilization. In response, we have added a summary table (Table 6) that presents all identified factors, the number of contributing studies, pooled odds ratios (ORs) with 95% confidence intervals (CIs), and heterogeneity (I²). This table provides a clear overview of the magnitude and consistency of associations across studies, enhancing the clarity and interpretability of our results and cited in the result also as table 6.

2. Geographical visualization

Revision action:

- If GIS mapping is not feasible, explicitly we add as not explained in the studies: This shows responsiveness even if a map cannot be added.

3. Funnel plot and Egger test with <10 studies

Mandatory revision:

- We add a clear cautionary statement: “Given that fewer than ten studies were included, funnel plots and Egger’s regression test have limited power to detect publication bias. Therefore, the absence of detected asymmetry should not be interpreted as evidence of no publication bias.”

4. Inconsistent effect sizes

Revision action:

- We standardize: Always report AOR (95% CI) and ensure pooled estimates match forest plots and text

5. Bayesian analysis had no results

- Bayesian meta-analysis was not performed due to the small number of studies and limited data for robust estimation

6. No comment on multiple comparisons

Revision action:

- Given multiple pooled associations were examined, findings should be interpreted cautiously, as the analysis was not adjusted for multiple comparisons.”

DISCUSSION

Major conceptual revision required (Reviewer is right)

1. We remove recommendations from early discussion for all paragraph

- Revision action:
   Early Discussion should: Interpret findings, Compare with literature and Explain mechanisms

2. Quantify “markedly low”

- Revision action:
  We replace with the actual pooled estimate as “Palliative care utilization was markedly low, with a pooled prevalence of 42%.”

3. Formatting inconsistencies

- Revision action:
  We standardize font, spacing, headings throughout Discussion.

4. Why Taiwan?

Reviewer is correct.

Revision action:

- Remove Taiwan comparisons
  Replace with regional comparators: Kenya, Uganda, Nigeria ,and Sub-Saharan Africa averages

5. “Good knowledge”

Revision action:

- We replace with: “Adequate knowledge”

Major critique: Discussion lacks analytical depth

Each paragraph should:

1. State the key finding
2. Explain why it occurs (mechanism/context)
3. Compare with regional/global evidence
4. Acknowledge uncertainty/heterogeneity

Only one consolidated recommendations section at the end was added

We believe that the revisions address all of the concerns raised and enhance the clarity, rigor, and readability of the manuscript. We thank Reviewer 4 again for their valuable insights, which have substantially improved the quality of our work.
