# Supplementary material for: Utilization of palliative care services and associated factors among cancer patients in Ethiopia: A systematic review and meta-analysis
Source: PLoS One. 2026 Mar 23;21(3):e0345299. doi: 10.1371/journal.pone.0345299 (PMC13008075; doi:10.1371/journal.pone.0345299)
Supplement: Attachment — Submitted filename: Response to Reviewers.docx [file pone.0345299.s009.docx]

Response to Reviewers

We sincerely thank the Editor and all reviewers for their careful re-review of our manuscript entitled “Utilization of Palliative Care Services and Associated Factors among Cancer Patients in Ethiopia: A Systematic Review and Meta-Analysis.” We appreciate the time and effort invested in providing constructive feedback. We have carefully revised the manuscript to address all comments raised, with particular attention to language quality, consistency, analytical rigor, and interpretation of findings. Detailed responses to each reviewer are provided below.

Reviewer #1

Comment: *“The authors responded to the raised issues and the revised version is much improved.”*

Author Response:

We sincerely thank Reviewer #1 for the positive evaluation and for acknowledging the improvements made in the revised manuscript. We appreciate your constructive feedback throughout the review process, which has significantly contributed to strengthening the clarity, methodological rigor, and overall quality of the manuscript.

Reviewer #4

Comment: *“Still disappointed with this revision 2. Glaring grammar issues, changes in font as things appear copy and pasted. Repeated mistakes notable with excessive comma usage, conjoining sentence structure and excessive listing without actually narrowing down points for the reader. The discussion also has heavy definitive statements forgetting the statistics remain very low quality given the paucity of data and high heterogeneity. I would like to remind the authors to refrain or avoid definitive statements especially around the data when describing what is being found.”*

Author response

We thank Reviewer #4 for the detailed and critical feedback. We acknowledge the concerns raised and have undertaken substantial and systematic revisions to address each issue comprehensively. Our responses are outlined below.

1. Grammar, sentence structure, and punctuation issues

Reviewer concern:
Glaring grammar issues, excessive comma usage, sentence conjoining, and overly long lists.

Author Response:

We fully acknowledge this concern and have thoroughly revised the entire manuscript for language quality. Specifically:

- The manuscript was line-by-line edited to correct grammatical errors, punctuation misuse (especially excessive commas), and sentence fragments.
- Long and conjoined sentences were split into shorter, clearer sentences to improve readability.
- Excessive listing was reduced and synthesized, with key points consolidated into concise analytical statements.
- Redundant phrases and repetitive constructions were removed throughout the manuscript.

2. Font inconsistency and formatting issues

Reviewer concern:
Changes in font suggesting copy-and-paste errors.

Author Response:
We agree with this observation and have corrected all formatting inconsistencies. Specifically:

- The entire manuscript was reformatted to ensure uniform font type, size, and spacing.
- All copied sections (tables, figures, references, and in-text citations) were standardized according to the journal’s formatting requirements.
- Headings, subheadings, and figure captions were harmonized for visual and structural consistency.

3. Overly definitive statements despite low-quality evidence and high heterogeneity

Reviewer concern:
Use of strong, definitive language despite high heterogeneity and limited data quality.

Author Response: We appreciate this important methodological reminder and have substantially revised the tone and interpretation of our findings. Specifically:

- All definitive and causal language was replaced with cautious, probabilistic, and interpretive wording
- Statements were explicitly contextualized within the limitations of cross-sectional designs, high heterogeneity (I² > 90%), and limited number of studies.
- We emphasized that pooled estimates are indicative rather than definitive national values, explicitly stating this in both the Results and Discussion sections.
- The GRADE certainty assessment was highlighted more clearly, noting that the overall certainty of evidence ranged from low to very low.

4. Interpretation of heterogeneity and data limitations

Reviewer concern:
Failure to adequately reflect the implications of high heterogeneity and data scarcity.

Author Response:
This concern has been carefully addressed. We now:

- Explicitly state that substantial heterogeneity limits generalizability.
- Clarify that observed variations are likely due to differences in outcome definitions, service availability, study settings, and patient populations.
- Avoid over-interpretation of subgroup analyses and state clearly when findings did not reach statistical significance.
- Added explicit cautionary statements regarding multiple comparisons and potential type I error.

INTRODUCTION

Comment 1

Remove “with” before “70% of these deaths…”

Author Response:
The unnecessary word “with” was removed to correct grammatical flow and sentence clarity.

Comment 2

Excessive commas noted in the line “Despite its proven…”

Author Response:
This sentence was restructured to reduce comma overuse and improve readability. The sentence was shortened and simplified while preserving its meaning.

Comment 3

Revise line “these challenges are particularly…” Excessively long and unnecessary commas

Author Response:
The sentence was rewritten into a concise structure, eliminating excessive commas and improving logical flow.

Comment 4

Revise paragraph 4. Excessive commas and excessive listing of challenges

Author Response:
We agree that over-listing reduced readability. This paragraph was substantially revised by:

- Grouping related barriers (e.g., workforce, access, awareness) into broader thematic categories
- Removing repetitive lists
- Improving narrative flow and analytical cohesion

This revision enhances clarity while preserving the importance of key factors.

Comment 5

Should “international partners” be a common “i”?

Author Response:
Yes. “International partners” was corrected to lowercase, as it is not a proper noun.

Comment 6

Revise line “Over the past two decades”. Same problem as above

Author Response:
This sentence was rewritten to avoid excessive listing and comma overuse. Information was synthesized into a coherent summary statement rather than a list.

Comment 7

Should “National health sector” be a common “n”?

Author Response:
Yes. The phrase was corrected to “national health sector” for grammatical consistency.

Comment 8

What gap is highlighted? Helpful

Response:
To clarify the research gap, we explicitly stated that:

- Existing evidence is fragmented
- There is no prior pooled national estimate
- Determinants have not been consistently synthesized

This gap is now clearly articulated in the final paragraph of the Introduction.

METHODS

Comment 1

Excessive use of “and” in “Aim of the study”

Author Response:
The sentence structure was revised to reduce repetitive use of “and.” Objectives were reorganized for clarity and conciseness.

Comment 2

Inconsistent capitalization of “palliative care”

Author Response:
We standardized terminology throughout the manuscript. “Palliative care” is now consistently written in lowercase unless appearing at the beginning of a sentence or in a title.

Comment 3

Why is “Search strategy” in a different font?

Author Response:
This was a formatting error introduced during revision. Font type and size were standardized.

Comment 4

Different fonts and irregular spacing in the Methods section

Author Response:
All subheadings and paragraphs were reformatted to ensure:

- Uniform font
- Consistent spacing
- Proper alignment between headings and text

Comment 5

Why are Funnel plot and Egger’s test mentioned again?

Author Response:
Redundant references were removed. Funnel plots and Egger’s test are now described only once in the Statistical Analysis subsection to avoid repetition.

RESULTS

Comment 1

Too many percentages listed consecutively in utilization rate paragraph

Author Response:
The paragraph was rewritten to:

- Present utilization as a range
- Highlight key extremes
- Avoid listing multiple percentages consecutively

This improves narrative clarity while preserving data accuracy.

Comment 2

Inconsistent font before Discussion heading

Author Response:
Formatting inconsistencies were corrected and fonts standardized.

DISCUSSION

Comment 1

Irregular fonts throughout the discussion

Author Response:
The Discussion section was fully reformatted to ensure consistent font style and spacing.

ADDITIONAL COMMENTS

Comment 1

Clarify that pooled prevalence (42%) is descriptive due to high heterogeneity

Author Response:
We explicitly stated that the pooled prevalence should be interpreted as a descriptive aggregate estimate, not a definitive national value, due to substantial heterogeneity.

Comment 2

P = 0.00 is not meaningful

Author Response:
All instances of “p = 0.00” were removed. Statistical significance is now reported appropriately using confidence intervals and contextual interpretation.

Comment 3

Ensure predictors are interpreted as associations, not causal

Author Response:
All predictors are now explicitly described as associations. Causal language was removed, and findings are interpreted correlationally

Comment 4

More interpretation needed for AOR > 5 for gender

Author Response:
We expanded the discussion to interpret this strong association by considering:

- Gender roles and decision-making power
- Financial autonomy
- Healthcare access differences
- Sociocultural norms

We also emphasized cautious interpretation given the small number of studies. We appreciate Reviewer #4’s detailed and technically rigorous feedback. All comments were carefully addressed through substantive language revision, formatting correction, and improved analytical interpretation, substantially strengthening the manuscript’s clarity, balance, and scientific rigor.
