# Supplementary material for: Utilization of palliative care services and associated factors among cancer patients in Ethiopia: A systematic review and meta-analysis
Source: PLoS One. 2026 Mar 23;21(3):e0345299. doi: 10.1371/journal.pone.0345299 (PMC13008075; doi:10.1371/journal.pone.0345299)
Supplement: Attachment — Submitted filename: Response to Reviewer.docx [file pone.0345299.s010.docx]

**Response to Reviewer #4**

We sincerely thank Reviewer #4 for the careful re-evaluation of our manuscript and for the constructive comments that have helped us further strengthen the methodological rigor, clarity, and reporting quality of our study. Below, we respond to each comment point-by-point and describe the corresponding revisions made in the manuscript.

**Comment 1: “Need more clarification why the methodology included pooling given such high heterogeneity.”**

**Author Response:**

We appreciate this important methodological concern. In the Statistical Analysis and Results (Pooled prevalence section), we added clarification explaining:

- Why pooling was performed despite high heterogeneity.
- That pooled estimates are interpreted as descriptive aggregates, not definitive national values.
- That REML random-effects modeling with Hartung–Knapp adjustment was used to account for between-study variability.
- That sensitivity and subgroup analyses were conducted.

In the Results section we insert as “Given the substantial heterogeneity observed across studies, this pooled estimate should be interpreted as a descriptive aggregate rather than a definitive prevalence.”

In the Statistical Analysis we insert as “A random-effects model using REML estimation was selected a priori due to anticipated clinical and methodological heterogeneity. Pooled estimates were interpreted cautiously, given high I² values.”

We clarified the rationale for pooling under high heterogeneity, explicitly justified the use of a random-effects REML model, and emphasized cautious interpretation of pooled estimates as descriptive summaries rather than precise national prevalence values.

**#Comment 2: “Explain/Remove/Address the significance of the Z Test given it adds little meaning.”**

**Author Response:**

We agree that the Z-test in prevalence meta-analysis provides limited interpretive value. We removed emphasis on the Z-test from the Results section. We retained it only where automatically generated but removed interpretative discussion. We clarified that statistical inference focuses on confidence intervals rather than Z statistics. We removed interpretative emphasis on the Z-test and now focus on confidence intervals and between-study variability, which are more meaningful in prevalence meta-analysis.

**#Comment 3: “Need more rigorous understanding of what is causing the heterogeneity. Given the geography did not explain it.”**

**Author Response:**

We appreciate this critical comment. we added in the Discussion, we expanded interpretation of heterogeneity sources: “The high heterogeneity likely arises from differences in outcome definitions, institutional capacity, urban–rural disparities, study populations, and availability of Palliative care services.” We also clearly stated that geographic subgrouping alone did not explain heterogeneity.

We expanded the Discussion to provide a more comprehensive interpretation of potential clinical, methodological, and contextual drivers of heterogeneity beyond geographic region.

**#Comment 4: “Why pool two studies?” (Gender analysis)**

**Author Response:**

We agree that pooling two studies requires caution. In the gender results section: “Because the pooled analysis was based on only two studies, assessment of publication bias using funnel plots or statistical tests was not performed.” In Discussion and Limitations: “The gender estimate is based on only two studies and should therefore be interpreted cautiously.” We retained the pooled estimate for transparency but clearly acknowledged its limited robustness and interpret it cautiously in both Results and Limitations.

**#Comment 5: “Explain the Tau². No interpretation of this value.”**

**Author Response:**

We added interpretation of Tau² In the Sensitivity Analysis section: as “Tau² represents the estimated between-study variance under the random-effects model, reflecting the magnitude of true heterogeneity beyond sampling error.” We also clarified that Hartung–Knapp adjustment incorporates Tau² uncertainty.

\#**Comment 6: “Consider meta-regression to help strengthen the analysis.”**

**Author Response:**

We carefully evaluated this suggestion. However: Only 9 studies were included, most predictors were reported in ≤3 studies, and Meta-regression requires ≥10 studies for stable estimation. We added in the In Limitations: “Meta-regression was not performed due to the small number of included studies, which would limit statistical power and produce unstable estimates.”

We considered meta-regression but did not perform it due to insufficient number of studies, and this limitation is now explicitly acknowledged.

**#Comment 7: “Hartung-Knapp mentioned however how did it affect your results?”**

**Author Response:**

We expanded explanation. We added In Statistical Analysis: “Hartung–Knapp adjustments were applied to produce more conservative confidence intervals, particularly important given the small number of studies and high heterogeneity.”

We clarified that this results in wider, more robust CIs. We clarified that Hartung–Knapp produced more conservative confidence intervals and improved robustness under high heterogeneity and small sample meta-analysis.

**#Comment 8: “How did you correct for multiple testing with the comparisons for the predictors?”**

**Author Response:**

We agree multiple comparisons increase Type I error risk. Given the exploratory nature and small number of pooled predictors, we did not formally apply Bonferroni correction. We added In Results: “Given the analysis of multiple factors, results should be interpreted cautiously, as the risk of type I error increases with multiple comparisons.” In Limitations: “No formal correction for multiple comparisons was applied due to the exploratory nature of the factor analyses.”

We clarified the absence of formal multiple testing correction and emphasized cautious interpretation.

**$Comment 9: “Discussion had run-on sentences and multiple comparisons of listing other countries, consider revising.”**

**Author Response:**

We thoroughly revised the Discussion by Split long sentences, reduced excessive listing of countries, focused on conceptual interpretation instead of country-by-country repetition, and Improved grammar and clarity throughout. The Discussion has been substantially edited for clarity, structure, and conciseness, with removal of run-on sentences and improved readability.

\#**Comment 10: “Ensure limitation encompass the issues you had faced doing the research eg. Gender estimates with 2 studies.”**

**Author Response:**

We expanded the Limitations section to include: High heterogeneity, Cross-sectional design, Urban hospital bias, small number of studies for predictors, Gender estimate based on only two studies, Lack of meta-regression, and Multiple testing concerns. The Limitations section has been strengthened to comprehensively reflect methodological constraints.

\#**Comment 11: “Minor formatting issues still present with missing spaces in the values and dash vs hyphen used.”**

**Author Response:**

We carefully proofread the entire manuscript and corrected: Missing spaces (e.g., “2.49(CI:” → “2.49 (95% CI:”), Hyphen vs en-dash consistency (95% CI: 30%–54%), Standardized statistical formatting, Figure labeling consistency, and removed duplicated PROSPERO number inconsistency. All formatting inconsistencies, spacing errors, and dash usage issues have been corrected throughout the manuscript.

We sincerely thank Reviewer #4 for the detailed and methodologically insightful comments. The manuscript has been substantially strengthened through all given comments and suggestion. We believe the revised manuscript now demonstrates stronger methodological transparency, clearer statistical justification, and improved scientific rigor.
